# How processing choices effect repeatability in BOLD–CVR imaging

**DOI:** 10.1177/0271678X261420026

**Published:** 2026-04-11

**Authors:** Gustav Magnusson, Alex A Bhogal, Charalampos Georgiopoulos, Gunnar Cedersund, Lovisa Tobieson, Maria Engström, Anders Tisell

**Affiliations:** 1 Department of Health, Medicine and Caring Sciences, Linköping University, Linköping, Sweden; 2 Center for Medical Image Science and Visualization (CMIV), Linköping University, Linköping, Sweden; 3 Translational Neuroimaging Group, Center for Image Sciences, University Medical Center Utrecht, Utrecht, The Netherlands; 4 Diagnostic Radiology, Department of Clinical Sciences, Medical Faculty, Lund University, Lund, Sweden; 5 Department of Biomedical Engineering, Linköping University, Linköping, Sweden; 6 Department of Biomedical and Clinical Sciences, Linköping University, Linköping, Sweden; 7 Clinical Department of Neurosurgery in Linköping, Region Östergötland, Linköping, Sweden; 8 Clinical Department of Medical Radiation Physics, Region Östergötland, Linköping, Sweden

**Keywords:** BOLD–MRI, breath-hold, cerebrovascular reactivity, CO_2_ inhalation, resting-state

## Abstract

Cerebrovascular reactivity (CVR) is increasingly recognized as a valuable clinical biomarker, making accurate, and reliable quantification essential, particularly in the absence of a gold-standard reference. However, both the acquisition and processing of CVR data are influenced by numerous methodological factors, including imaging technique, sequence parameters, vascular paradigm, and data processing strategies. These complexities can be daunting for newcomers and hinder methodological consistency across studies. To support both novice and experienced researchers, we systematically evaluated how different processing strategies affect CVR map repeatability, quantified using spatial intraclass correlation, across multiple vascular paradigms. Twenty-four healthy volunteers underwent BOLD–CVR imaging using CO_2_ inhalation, breath-hold, and resting-state paradigms, each repeated in a test–retest setup. We found that optimal processing choices varied across paradigms and interacted in non-trivial ways. For example, the inclusion of motion-confounds and the application of temporal filtering require careful consideration, as they can introduce substantial collinearity in the regression model and reduce repeatability. We summarized these findings into practical insights to guide researchers in making sound methodological choices and promote consistency within the field.

## Introduction

Cerebrovascular reactivity (CVR) refers to the ability for blood vessels to dilate and constrict in response to vasoactive stimulus. CVR mapping has become increasingly important tool in both clinical and research settings, particularly for assessing vascular health in conditions such as stroke, brain tumors, small vessel disease, and neurodegenerative diseases.^1^

CVR is typically measured using magnetic resonance imaging (MRI) based techniques in combination with a stimulus paradigm to evoke a vascular response, referred to here as a vascular paradigm. A commonly used MRI sequence is blood-oxygen-level-dependent (BOLD) imaging, which is also used in functional MRI (fMRI), due to its high temporal resolution and signal-to-noise ratio (SNR).^2^ The most frequent vascular paradigms are carbon dioxide (CO_2_) inhalation and breath-holding (BH).^1^ CO_2_ inhalation increases blood CO_2_ partial pressure (PCO_2_) and lowers blood pH, resulting in vasodilation, and provides a well-controlled and reproducible stimulus, though it requires specialized delivery equipment.^3^ BH also results in increased PCO_2_ and is, by contrast, more accessible but introduces greater variability due to differences in subject compliance.^4^ An alternative approach, resting-state (RS) CVR, avoids external stimuli altogether, and estimates CVR from spontaneous fluctuations in respiration and end-tidal CO_2_ (ET-CO_2_).^5^ However, this method assumes that sufficient vascular responses occur at rest, which may limit its sensitivity to detect underlying pathophysiology or blood redistribution leading to vascular steal, a local reduction in blood flow caused by increases elsewhere and an important marker of vascular impairment.^6^

While different vascular paradigms are generally assumed to probe the same underlying physiology, the ways in which they elicit a vascular response differ. Consequently, the same data processing strategies, such as the degree of spatial smoothing, temporal filtering, inclusion of motion-confounds, and choice of regressor, may not be equally suitable across paradigms, and different parameters might interact in complex ways.

Individual comparisons, such as CO_2_ inhalation versus BH,^7^ BH versus RS,^8^ and CO_2_ inhalation versus RS,^9^ have previously been investigated, however no study (to the authors’ knowledge) have simultaneously evaluated more than two paradigms within the same cohort or examined how optimal processing strategies may differ between them.

Several studies have examined the selection of regressor for CVR mapping. In BH-based CVR this has been done to mitigate the effect of low-patient compliance^4^ or to compensate for the absence of high-quality CO_2_ recordings.^10,11^ Other work has addressed regional variability in cerebrovascular dynamics by accounting for differences in delay and/or fitting a hemodynamic response function (HRF).^12–15^ Data-driven approaches have also been proposed, either by deriving a regressor from the mean signal within a predefined region^16^ or by refining an initial regressor (e.g. a CO_2_ trace) by finding highly correlated imaging signals and aligning them to maximum correlation to produce a new regressor.^17^

A smaller number of studies have systematically examined other aspects of CVR data processing. For example, Liu et al. investigated optimal temporal filtering of RS signals to improve the spatial correspondence between RS-CVR and CO_2_-CVR maps,^9^ while Moia et al. employed independent component analysis (ICA) to remove motion-related signals while preserving CO_2_-related fluctuations.^18^

There also exists a rich body of fMRI studies examining methodological choices. However, it remains unclear whether these findings are directly transferable to CVR imaging, particularly given that optimal processing strategies within fMRI, such as degree of spatial smoothing and/or removal of motion influence, can depend on the specific experimental design and research question.^19–22^

Although many CVR processing approaches exist, the literature offers little systematic, evidence-based guidance to inform processing choices and ensure reproducible results.

In this study, we sought to address this gap by systematically comparing BOLD-based CVR derived from CO_2_ inhalation, BH, and RS within a single cohort of healthy subjects. Our primary aim was to evaluate how widely used processing steps, including spatial smoothing, linear detrending, temporal filtering, and inclusion of motion-confounds, affect the repeatability of CVR maps across vascular paradigms. We found that while several choices, such as spatial smoothing, exerted broadly similar effects across paradigms, others, most notably temporal filtering and motion-confounds, showed more variable impacts and strong interactions with one another.

By presenting our findings within a cohesive framework, we provide actionable, data-driven recommendations to improve CVR repeatability and to guide methodological decision-making, particularly for researchers entering the field.

## Materials and methods

### Subjects

All subjects gave written informed consent in accordance with the Declaration of Helsinki. The study was approved by the Swedish Ethical Review Authority (ref: 2021-04825). Before participation, subjects completed a health form to screen for factors affecting cerebrovascular function or task compliance. Inclusion required good general health and no history of brain surgery, neurological/psychiatric disorders, cognitive impairment, or substance abuse. Subjects reported prescription drug use; only those on psychoactive medications were excluded. Additional exclusions included diagnosed pulmonary disease and regular smoking.

### Procedure

Subjects underwent MRI examinations of BOLD–CVR using three vascular paradigms: fixed CO_2_ inhalation, BH, and RS, each repeated in a test–retest design. These data were subsequently used to evaluate how processing choices affect repeatability and to gain insights into CVR image generation, more details below.

After completing the MRI session, subjects rated their discomfort for each vascular paradigm, as well as their general discomfort from wearing a facemask (see below) and from being inside the MRI scanner. Discomfort was rated on a scale from “None” to “Extreme.” For the CO_2_ paradigm, subjects additionally reported any experienced symptoms (e.g. dizziness, anxiety). Differences in discomfort ratings were assessed using Friedman’s test. Significant results were followed by Wilcoxon signed-rank tests with Bonferroni correction. Further details are provided in Supplementary Material—Discomfort.

We also evaluated differences in head motion across paradigms by comparing mean framewise displacement (FD) during the MRI acquisitions. Pairwise comparisons were conducted using paired *t*-tests with Holm correction for multiple comparison. Further information is provided in Supplementary Material—Head motion.

### Data acquisition

BOLD–CVR was measured on a 3 T MRI scanner (MAGNETOM Prisma; Siemens Healthineers, Forcheim, Germany), under one of three vascular paradigms: fixed CO_2_ inhalation, BH, or RS. Each 5-min run was block-randomized (block size = 6). To assess test–retest repeatability, each paradigm was repeated after a 3–4 min break, during which subjects exited the scanner briefly.

**Figure 1.**
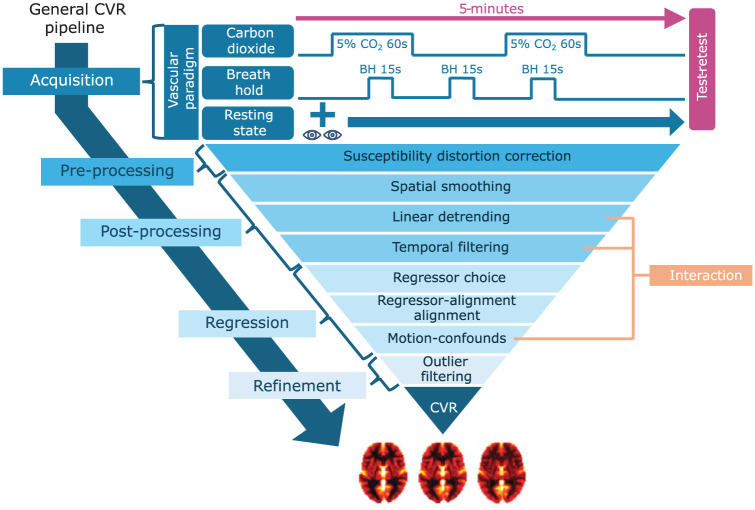
Overview of the design parameters selected for in-depth analysis, organized within a generalized CVR processing pipeline. The pipeline includes key stages: data acquisition, pre-/post-processing, regression analysis, and refinement. Parameters examined include vascular paradigm (with the specific stimulus protocols used in this study illustrated), susceptibility distortion correction, spatial smoothing, regressor choice, and outlier filtering. Linear detrending, temporal filtering, and motion-confounds were assessed jointly to explore their interaction effects. For a full list of all design parameters and their tested values, see Supplementary Table S1.

For the CO_2_ paradigm, we used our in-house iCO_2_ CVR system,^23^ involving a 30 s baseline, two 60 s blocks of 5% CO_2_, each followed by 75 s of room air. BH instructions, tailored to subjects’ breathing pace,^24^ were displayed using PsychoPy2,^25^ a screen and a projector. The BH task included three 15 s end-expiratory holds, interleaved with 45 s of paced breathing, plus 60 s paced breathing before and after. Compliance with the BH task was verified post-acquisition by visual inspection of the respiratory traces recorded by the iCO_2_ CVR system. During RS, subjects fixated on a cross without task or paced breathing. The stimulus protocols are shown in top part of Figure 1. To aid compliance, shorter practice versions of CO_2_ and BH were conducted pre-scan.

Subjects wore a facemask (Mask 7450 V2; Vyaire, Chicago, USA) connected to an MRI-conditional monitor (Expression MR400; Philips, Orlando, USA) throughout (excluding breaks) to deliver CO_2_ and sample ET-CO_2_ (sample rate: 1 Hz). For BH, subjects exhaled before and after each hold to aid interpolation, assuming linear ET-CO_2_ increase. An issue was that the MR400 only provided calculated ET-CO_2_, not full traces, which occasionally resulted in missing post-BH measurements. These missing values were imputed using data from the subject’s other BH trials. Additional details on handling of CO_2_ data is provided in the Supplementary Material—CO_2_ sampling.

Alongside functional T2*-weighted BOLD images, one T1-weighted MPRAGE image was acquired for registration and two (one per test/retest) dual-echo fieldmaps for susceptibility distortion correction (SDC). Acquisition parameters are listed in Supplementary Table S2.

### CVR image and signal processing

Below, we describe the processing strategies, or “design parameters,” considered in this study, including both parameters left untested and those systematically varied. All strategies were applied identically across the vascular paradigms used for CVR imaging. For each tested parameter, we report the range of values examined as well as the default setting used during evaluation. When presenting paradigm-independent results, we choose to focus on the CO_2_ paradigm, effectively treating it as the default paradigm. A complete overview of all parameters, including their tested levels and default values, is provided in Supplementary Table S1.

BOLD data were converted to BIDS-compliant NIfTI using DCM2Bids v3.1.1,^26^ and preprocessed with fMRIPrep v23.2.0a3,^27^ including motion correction, SDC, MNI normalization (MNI152NLin2009cAsym), and FreeSurfer v7.3.2^28^ reconstruction. To assess the impact of SDC, we additionally ran fMRIPrep without including the fieldmaps (effectively disabling SDC), default: SDC enabled.

Further processing used a custom Python v3.8 framework with Nilearn v0.10.4, SciPy v1.14.1, Pandas v2.2.3, and NumPy v2.1.1.^29–33^ Brain voxel-masking used Nilearn’s compute_epi_mask (“EPI-mask”; default) or fMRIPrep’s provided mask (“brain-mask”). Gaussian smoothing was applied using a variable full width at half maximum (fwhm; fwhm 0–9 mm; default: 5 mm; 0 mm = no smoothing).

Both voxel- and region-based analyses were conducted using Nilearn’s NiftiMasker/NiftiLabelsMasker. Region-based analysis used aparc + aseg parcellation^34^; voxel-wise analysis was default. BOLD and confound timeseries (from fMRIPrep) were upsampled (factor: 1–4; default: 2), as was ET-CO_2_, to match the temporal resolution. Polynomial linear detrending (degree 0–3; default: 1; 0 = no detrending) and low-pass temporal filtering (cutoff: 7.275–465.6 mHz, ∞; default: 116.4 mHz; ∞ = no filter) was applied to all timeseries. BOLD signals were scaled to percent signal change (PSC) using variable baseline strategies (see Supplementary Table S1; default: overall-mean). Global signal was PSC-scaled after summing unscaled signals. ET-CO_2_ baselines were subtracted using the same approach. To correct for acquisition delays, ET-CO_2_ was aligned to global signal via cross-correlation (±15 s). The first 15 s were then discarded to ensure steady-state.

CVR, defined as %BOLD change per unit change in regressor, was estimated via linear regression using BOLD timeseries as the dependent variable and either ET-CO_2_ (default for CO_2_/BH) or global signal (default for RS) as regressor. To account for physiological delay, the regressor was optionally shifted to the point of absolute maximum correlation with individual BOLD timeseries using cross-correlation and variable max-delay (±0–60 s, ∞; default: 0 s = no alignment). Timeseries were then downsampled to original BOLD sampling rate (0.878 s). Motion-confounds (24 timeseries; *x*, *y*, *z* translation and rotation plus their temporal derivatives, and squared terms) were included in the regression model if their absolute correlation with the main regressor was below a variable threshold (0–1; default: 0 = no motion-confounds).

To ensure robust repeatability estimates, quantified in this study using correlation-based metrics (see below), which are highly sensitive to extreme values, we removed outlying CVR estimates using a median absolute deviation (MAD) filter with a *z*-corrected threshold of 10 (approximately equivalent to excluding values more than 10 standard deviations from the mean under a normal distribution). While this approach was appropriate for our dataset of healthy volunteers, caution is warranted when applying outlier filtering more broadly, as overly aggressive thresholds may inadvertently remove physiologically meaningful signal in clinical or heterogeneous populations.

As a final step, we evaluated additional techniques for refining the CVR maps. First, we assessed several quality metrics for identifying low-quality voxels, including overall model fit (coefficient of determination, *R*^2^), regressor-specific fit (*t*-value), susceptibility-related distortion (fieldmap magnitude (fmap)), and temporal signal-to-noise ratio (tSNR; mean signal divided by its standard deviation, computed on raw BOLD data). For each metric, the lowest-performing 10% of voxels were removed; by default, no quality-based exclusion was applied. We then examined normalization strategies in which CVR maps were scaled to the mean value within gray matter (GM), white matter (WM), cerebrospinal fluid (CSF), or the whole-brain (WB), based on tissue segmentations obtained with FSL FAST (via fMRIPrep). The default was to apply no normalization. Analyses could also be restricted to a specific tissue of interest (default: WB).

### Analysis

The primary outcome measure was test–retest repeatability of CVR maps, measured using voxel-wise intraclass correlation ICC(C,1).^35^ We assessed how different design parameters, as detailed above, influenced repeatability across the three vascular paradigms: CO_2_ inhalation (from here on referred to as CO_2_), BH, and RS.

The analysis had two stages. First, we conducted a univariate sensitivity analysis: each parameter was varied individually (others held at default; see Supplementary Table S1), and repeatability of CVR maps were calculated.

Based on these results, key parameters (see Figure 1) were selected for a second, in-depth analysis. We focused on:

- *SDC:* Compared voxel masks with/without correction to assess spatial alignment.
- *Smoothing:* Analyzed how spatial smoothing influenced repeatability and subject/tissue contrast.
- *Linear detrending, temporal filtering, motion-confounds:* Explored combined and interacting effects using Fourier analysis and regressor–confounds collinearity.
- *Regressor repeatability:* Assessed how regressor stability related to CVR repeatability.
- *Regressor alignment:* Evaluated the impact of aligning the regressor to individual BOLD signals.
- *Quality metrics:* Investigated the relationship between tSNR and CVR across tissue types.

#### Univariate sensitivity analysis of design parameters

For each vascular paradigm, we perturbed individual parameters while keeping others fixed (defaults in Supplementary Table S1). Test–retest repeatability was quantified using ICC(C,1). Effects were evaluated with a repeated-measures Anova (AnovaRM) model after Fisher *z*-transformation of ICC values, implemented in R v4.4.3^36^ using the function aov_ez from the package afex v1.5-0^37^ via the Python-R interface rpy2.^38^ Correction of *p*-values for non-sphericity was performed using the Huynh–Feldt (HF) method, which has been shown to be a robust approach under non-normality conditions^39,40^ and is recommended for smaller sample sizes and multiple within-subject measurements.^41^ When sphericity corrections could not be performed, due to factor having only two levels (sphericity not defined) or singularities in the covariance matrix, uncorrected *p*-values were reported and explicitly indicated. For each factor we report *F*-value, degrees of freedom, HF correction factor, corresponding *p*-value and partial effect sizes (η^2^).

This exploratory analysis aimed to identify patterns for future validation; therefore, reported *p*-values were not corrected for multiple comparison.

#### In-depth analysis of design parameters

##### Susceptibility distortion correction

To evaluate the impact of SDC, we examined averaged fieldmaps, thresholded at ±40 Hz (pixel-bandwith was ~30 Hz/pixel in the phase-encoding). Differences between EPI-masks (with vs without SDC) were tested using FSL Randomise v2.9^42^ with threshold-free cluster enhancement (TFCE) and α = 0.05.

##### Smoothing

We evaluated the effect of spatial smoothing on repeatability and on subject/tissue contrast across fwhm levels. Subject contrast was quantified using “differential correlation,” a metric used in fMRI to assess how uniquely statistical maps identify individual subjects.^43^ Briefly, voxel-wise Pearson correlation of CVR maps between runs were computed for all pairs of subjects and differential correlation was defined as the mean within-subject correlation minus the mean between-subject correlation. Tissue contrast was assessed by generating average CVR maps and extracting mean CVR values within GM, WM, CSF, and WB.

##### Interaction

We evaluated the combined impact of linear detrending, temporal filtering, and motion-confounds on test–retest ICC(C,1). Every parameter combination was tested and AnovaRM models, as described above, were used to assess main effects and interactions, again correcting from sphericity but not for multiple comparison.

We also analyzed qualitatively how confound–regressor collinearity affected repeatability, computing *R*^2^ with the regressor as the dependent variable. FFTs were used to assess how parameter combinations altered regressor spectra.

##### Optimization

Continuing on the analysis above for linear detrending, temporal filtering, and motion-confounds, we aimed to isolate each parameter’s unique effect by performing an “optimized perturbation” where each parameter was varied while others were fixed at optimal values (based on test–retest ICC(C,1)). As a sanity check, we repeated the optimized and univariate perturbation using voxel-wise Pearson correlation with a reference map, the vascular paradigm and parameter combination with the highest test–retest repeatability, instead of test–retest ICC(C,1).

We also examined whether amount of head motion (measured by average FD) correlated with improvement in test–retest ICC(C,1) when including motion-confounds in the linear regression model. Improvements were assessed relative to models without motion-confounds, using optimal linear detrending and temporal filtering parameters for each condition.

Finally, we computed voxel-wise quality metrics using optimal values for linear detrend order, low-pass cutoff frequency, and confound–regressor correlation thresholds (based on test–retest ICC(C,1)) for each vascular paradigm:

- Normalized CVR, scaled by the median CVR across voxels, subjects, and runs.
- Model fit, measured by *R*^2^.
- Regressor fit, measured by *t*-value.
- Average absolute CVR difference between test–retest, also scaled by the median CVR.
- Coefficient of variation (COV), defined as standard deviation divided by absolute mean (limited to 1 to avoid arbitrary large values when mean CVR is close to 0).
- Reliability, assessed using ICC(C,1) between test–retest and across subjects.

To avoid edge effects due to varying brain voxel-masks, all metrics above were restricted to voxels with a valid mask in at least 50% of subjects and runs.

##### Regressor

To assess the repeatability of the regressor itself, the timeseries underwent default linear detrending and temporal filtering. Test and retest regressors were aligned (±15 s) before computing ICC(C,1) between the two. Repeatability of ET-CO_2_ versus global signal regressors was compared using paired *t*-tests on *z*-transformed ICCs and compared with corresponding CVR map repeatability. We also compared mean WB-CVR between test–retest. Normality was assessed using the Shapiro–Wilk test, followed, when significant, by inspection of *Q*–*Q* plots. Effect sizes are reported using Cohen’s *d*.

##### Delay

We assessed how aligning regressors to individual BOLD signals affected repeatability. Specifically, we analyzed test–retest differences in CVR maps and investigated whether these differences could be attributed to the repeatability of estimated delays (measured as absolute delay differences), regressor–BOLD correlation, the maximum allowable delay, and the regressor’s autocorrelation function.

We also evaluated if applying a median filter to delay values (kernel size 3 × 3 × 3) prior to the CVR estimation, could improve CVR test–retest repeatability.

##### Quality metrics

Among the tested quality metrics used to improve CVR repeatability, we choose to focus on tSNR. To maintain measure independence, tSNR was computed from RS data (RS-tSNR) and CVR from CO_2_ inhalation (CO_2_-CVR). We compared them using scatterplots and averages maps across subjects. Mean values were also calculated within GM, WM, and CSF after within-subject standardization to enable comparison across tissues.

## Results

In total 25 subjects were recruited; however, one was later excluded due to difficulties with being inside of the MRI-scanner, leaving 24 subjects (four complete blocks of size 6) that completed the study. The median age was 26 (range: 18–52), and 63% of subjects (*n* = 15) were women. Results from the discomfort survey are provided in Supplementary Figure S3 and Table S3. No significant differences in discomfort were reported between the different vascular paradigms.

Head motion was highest during BH (mean FD = 0.14 mm), followed by CO_2_ (0.12 mm) and RS (0.11 mm). However, only the BH–RS comparison was statistically significant. For more information see Supplementary Material—Head motion.

All participants demonstrated good compliance with the BH task, as confirmed by inspection of the respiratory traces. In three measurements, the respiratory trace was accidently not saved; in these cases, compliance was instead verified by inspection of the global BOLD signal.

Supplementary Figure S5 and Table S4 present the results of the initial univariate sensitivity analysis. In the sections below, we focus on the in-depth analysis of design parameters illustrated in Figure 1, beginning with SDC.

### Susceptibility distortion correction

**Figure 2.**
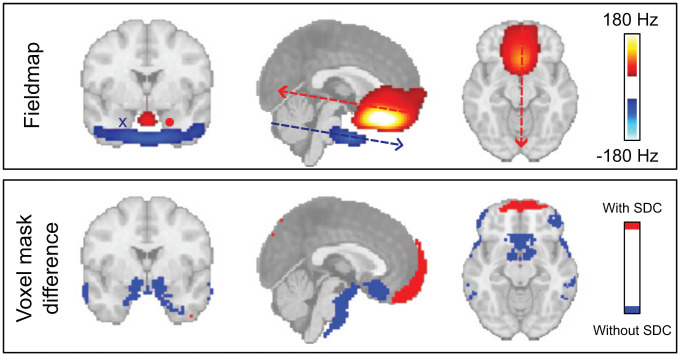
Effect of SDC. (top panel) Average fieldmap across subjects and runs, thresholded at ±40 Hz, highlighting areas with substantial field inhomogeneity, most prominently around the frontal sinuses and ear canals, and indicating the direction of voxel displacement along the phase-encoding (anterior–posterior) axis. (bottom panel) Regions showing significant differences (*p* < 0.05) in brain voxel-mask (EPI-mask) with and without SDC, illustrating the spatial extent of geometric distortions corrected by the SDC procedure and signal loss. These EPI-mask were based on BOLD data acquired during the CO_2_ paradigm. SDC: susceptibility distortion correction.

We observed pronounced positive field offsets near the frontal sinuses, while large negative distortions appear around the ear canals (Figure 2, top panel). In areas adjacent to these regions, we identified significant differences (*p* < 0.05) in voxel-wise brain masks with and without SDC (Figure 2, bottom panel). At the same time, we only saw moderate improvement in repeatability comparing CVR maps with and without SDC (Supplementary Figure S5), which reached significant levels for BH (*p* = 0.048; η^2^ = 0.16), but not for CO_2_ (*p* = 0.053; η^2^ = 0.15) or RS (*p* = 0.23; η^2^ = 0.06).

### Smoothing

**Figure 3.**
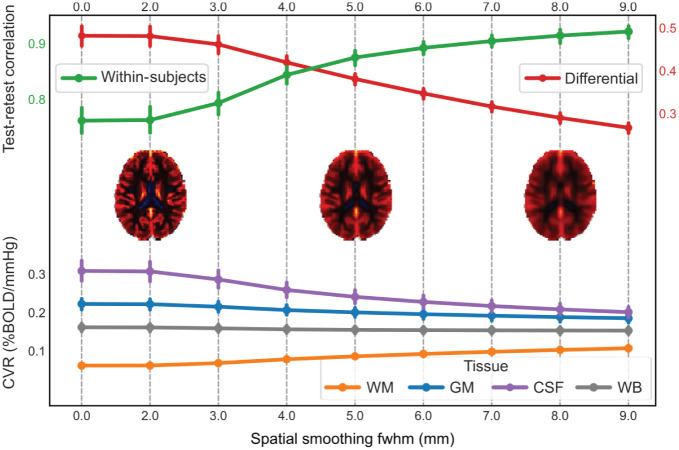
Effect of spatial smoothing on CVR map repeatability and tissue/spatial contrast for the CO_2_ paradigm. (top panel) Average test–retest Pearson correlation of CVR maps within the same subject (green graph) and differential correlation (average within-subject correlation minus average between-subject correlation; red graph) across spatial smoothing levels (0–9 mm fwhm). Within-subject correlation increases with smoothing, indicating improved repeatability, but at the same time the differential correlation reduces, indicating reduced subject-contrast. (middle row) Mean CVR maps at 2, 5, and 8 mm fwhm illustrate the progressive blurring of anatomical boundaries with increased smoothing. (bottom panel) average CVR values within different tissue types, WM, GM, CSF, and WB, as a function of fwhm. As smoothing increases, tissue-specific values converge toward the WB average, reflecting a loss of tissue contrast. Error bars represent 95% confidence intervals. WM: white matter; GM: gray matter; CSF: cerebrospinal fluid; WB: whole-brain.

We observed a clear increase in test–retest ICC(C,1) as the fwhm increases from 0 mm (no smoothing) to 9 mm (*p* < 0.001) for all vascular paradigms (η^2^ = 0.97, 0.91, 0.91 for CO_2_, BH, RS, respectively). However, the relative improvement in repeatability diminishes with higher smoothing levels (Supplementary Figure S5). At the same time there was a loss of subject and tissue contrast (Figure 3). Particularly we note that CVR in WM tends to be overestimated and in CSF underestimated.

### Interaction

For all paradigms the confound–regressor correlation threshold (η^2^ = 0.95, 0.83, 0.65 for CO_2_, BH, RS, respectively), low-pass cutoff frequency (η^2^ = 0.96, 0.95, 0.94) and their two-way interaction (sphericity uncorrected; η^2^ = 0.82, 0.73, 0.66) were significant (*p* < 0.001). The linear detrend order was significant for CO_2_ (*p* = 0.030; η^2^ = 0.14) and RS (*p* < 0.001; η^2^ = 0.54) but not for BH (*p* = 0.11; η^2^ = 0.09), and its interaction with low-pass cutoff frequency was significant for CO_2_ (*p* < 0.001; η^2^ = 0.20) but not for BH (*p* = 0.097; η^2^ = 0.07) or RS (*p* < 0.13; η^2^ = 0.07). Additionally, the interaction between linear detrend order and confound–regressor correlation threshold was significant in all paradigms (*p* < 0.001; η^2^ = 0.22, 0.25, 0.53), as was the three-way interaction among all factors (sphericity uncorrected *p* < 0.001; η^2^ = 0.16, 0.08, 0.07). In Supplementary Table S5, complete results from the AnovaRM analysis are given, including *F*-values and HF correction factors.

**Figure 4.**
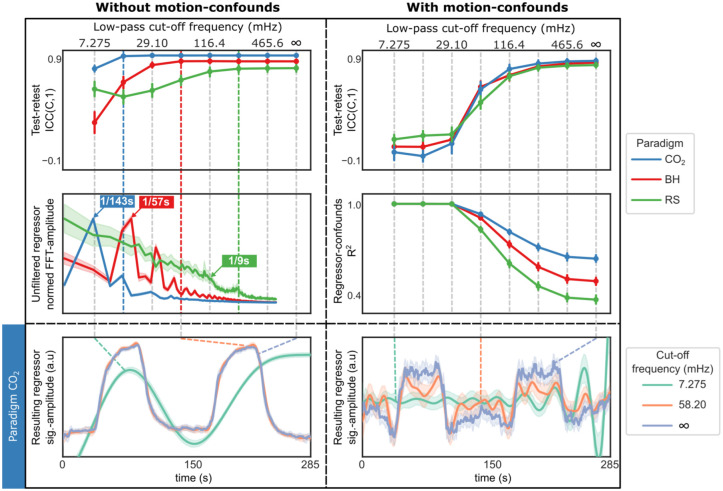
Interaction between temporal filtering and motion-confounds. (top row) Test–retest ICC(C,1) for CVR maps as a function of low-pass cutoff frequency for each vascular paradigm, (left) without motion-confounds (regressor-confound correlation threshold = 0.0), (right) with motion-confounds (regressor-confound correlation threshold = 1.0). Without motion-confounds, repeatability plateaus at different frequencies for the different vacular paradgims, indicated by colored dotted lines. With motion-confounds, repeatability improves only as cutoff frequency approaches infinity. (middle-left) In the frequency spectra of each (unfiltered) regressor timeseries, normed by the maximum mean amplitude, major components are evident for CO_2_ and BH; RS shows reduced power around 116.4 mHz. (middle-right) The collinearity (*R*^2^) between regressor and motion-confounds is near perfect at cutoff frequency below 29.10 mHz and reduces successively as the frequency increases. (bottom row) ET-CO_2_ regressors for CO_2_ paradigm are markedly different with and without motion-confounds, after filtering at different cutoff frequencies. Without motion-confounds (left), only the 7.275 mHz version (turquoise) deviates notably from the expected shape (two equal-sized 60 s blocks). With motion-confounds (right), only the unfiltered version (purple) retains the expected shape. Motion-confounds were regressed from the regressor for visualization only; in CVR regression analysis, they were included in the design matrix. Error bars represent 95% confidence intervals.

Without motion-confounds, CO_2_ and BH CVR map repeatability was largely insensitive to the exact low-pass cutoff frequency, if major regressor components were preserved (Figure 4, top-left and middle-left panels). RS showed a similar trend, though the regressor lacked clear spectral peaks and instead showed a decline in power around 116.4 mHz (~1/9 s). When motion-confounds were included, the regressor and confounds exhibited near-perfect collinearity for low-pass cutoff frequencies below 29.19 mHz, leading to poor repeatability across all vascular paradigms (Figure 4, top-right and middle-right panels). As the cutoff frequency increased, collinearity decreased, and repeatability improved. We also note that without motion-confounds, the regressor shape remains largely preserved except at the lowest cutoff (7.275 mHz; Figure 4, bottom left panel). When motion-confounds are included, however, both 7.275 and 58.20 mHz cutoffs distort the regressor substantially, while the unfiltered version retains the expected temporal profile (Figure 4, bottom right panel). The effect of linear detrending on repeatability was diminished when motion-confounds was included (Supplementary Figure S7, top panels). A likely explanation seems to be that the effect of linear detrending is to remove low-frequency components, however these are already removed when motion-confounds are included (Supplementary Figure S7, bottom panels).

### Optimization

Using optimized perturbed parameters, we found that the linear detrend order no longer had a significant effect on RS paradigm (*p* = 0.40; η^2^ = 0.04). Likewise, the confound-regressor correlation threshold no longer significantly affected paradigms BH (sphericity uncorrected *p* = 0.15; η^2^ = 0.07) or RS (sphericity uncorrected *p* = 0.74; η^2^ = 0.02). All other parameters remained statistically significant, see Supplementary Table S6 for summary.

When accounting for head motion, we observed that subjects with the highest amount of motion during BH (average FD > 0.2 mm) showed improved test–retest ICC(C,1) when motion-confounds were included in the linear regression model (Supplementary Figure S4). In contrast, for the CO_2_ and RS paradigms, amount of head motion did not correlate with changes in repeatability.

**Figure 5.**
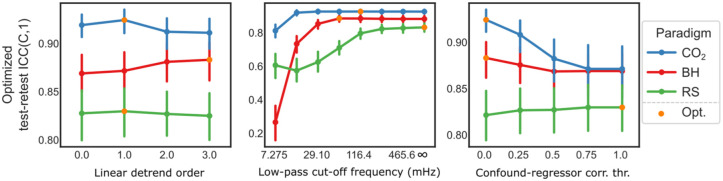
Optimized perturbation of design parameters. Each design parameter, linear detrend order, low-pass cutoff frequency, and confound–regressor correlation threshold, is perturbed individually. At each level, the other two parameters are held at their empirically optimal settings, defined by the highest mean test–retest ICC(C,1). The overall optimal combination for each vascular paradigm is highlighted with an orange marker. Error bars represent 95% confidence intervals.

Overall, the CO_2_ paradigm achieved the highest test–retest ICC(C,1) using the default settings: first-order detrending, a low-pass cutoff frequency of 116.4 mHz, and exclusion of motion-confounds. Consequently, this configuration was selected as the reference CVR map. Similar trends to those shown in Figure 5 were observed when calculating whole-brain voxel-wise Pearson correlations with this reference map instead of test–retest ICC(C,1); (Supplementary Figure S8). However, RS showed a local peak in correlation around low-pass cutoff frequency 232.8 mHz under the univariate perturbation analysis. Summaries of the optimized and univariate perturbed analyses using correlation with the reference CVR map are provided in Supplementary Tables S7 and S8, respectively.

Using the optimized processing combination shown in Figure 5 for each paradigm, we qualitatively observed that the average median-scaled CVR maps appeared similar across paradigms, as did the distribution of voxel-wise values (Supplementary Figure S9, top section). However, when comparing quality metrics (*R*^2^, *t*-value, CVR absolute difference, CVR COV, CVR reliability) the CO_2_ paradigm consistently outperformed the other two paradigms, with BH performing second best (Supplementary Figure S9, bottom sections).

### Regressor

For the CO_2_ paradigm, regressor repeatability was significantly higher for the ET-CO_2_ regressor compared to the global signal regressor (*p* = 0.0016; Cohen’s *d* = 0.73). No significant differences were observed for BH (*p* = 0.30; Cohen’s *d* = 0.21) or RS (*p* = 0.95; Cohen’s *d* = −0.012). In contrast, the corresponding CVR map repeatability showed significant lower repeatability (*p* < 0.001) using the ET-CO_2_ regressor across all paradigms (Cohen’s *d* = −0.79, −1.0, −2.3 for CO_2_, BH, RS, respectively). Supplementary Figure S10 shows a graphical representation of above results and Supplementary Table S9 a summary of the statistical tests.

Additionally, across all paradigms, we observed a small but consistent reduction in whole-brain average CVR values between test and retest sessions using the ET-CO_2_ regressor, however only significant for CO_2_ paradigm (*p* = 0.036, Cohen’s *d* = −0.46). See Supplementary Table S10 for summary.

### Delay

**Figure 6.**
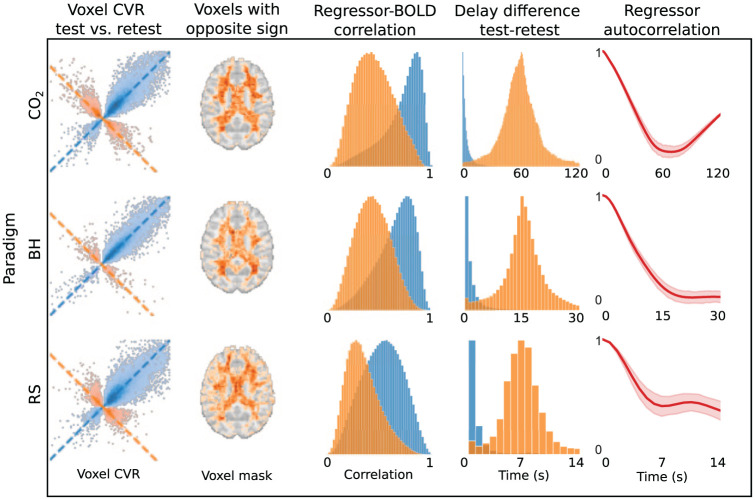
Effects of voxel-wise regressor alignment. (column 1) Voxel-wise CVR values from a representative subject, comparing test (*x*-axis) and retest (*y*-axis) for the CO_2_, BH, and RS paradigms. Blue points indicate voxels with consistent sign and values along the identity line (*x* = y), while orange points indicate sign reversals and values along the anti-diagonal (*x* = −*y*), indicating anti-correlation. (column 2) Spatial distribution of anti-correlated voxels (orange), averaged across subjects and thresholded at 10% occurrence, primarily located in deep white matter. (column 3) Distribution of regressor–BOLD correlation coefficients for blue and orange voxel groups (scaled by peak density), highlighting weaker signal in anti-correlated voxels. (column 4) absolute delay differences between test and retest for each group, and (column 5) regressor autocorrelation structure, showing a plateau, or minima, corresponding to delay were orange voxels pile up in column 4. Delay bounds for alignment were set to: CO_2_ = unbounded, BH = ±15 s, RS = ±8 s.

Aligning the regressor to account for voxel-wise temporal delays (Supplementary Figure S5) reduced CVR map repeatability across all paradigms (*p* < 0.001; η^2^ = 0.83, 0.88, 0.86 for CO_2_, BH, RS, respectively). We found that the regressor alignment introduced a subset of voxels showing anti-correlated CVR values, similar amplitude but different signs, between test and retest (Figure 6, column 1), primarily located in deep white matter (Figure 6, column 2). These voxels exhibited lower regressor–BOLD correlations (Figure 6, column 3), indicating poor model fit. The absolute delay differences between test and retest also clustered around paradigm-specific times (Figure 6, column 4), which corresponded to inflection points in the corresponding regressor’s autocorrelation function (Figure 6, column 5). This suggests that instability in delay estimation, particularly in regions with low signal, may drive the observed decrease in repeatability following alignment.

Given that negative CVR has often been associated with vascular steal effects, although other physiological and non-physiological mechanisms may also contribute, we will refer to these anti-correlated voxels introduced by regressor alignment as “fake steal.” We also observed that applying a median filter to delay values prior to CVR estimation, largely removed the anti-correlated voxels (Supplementary Figure S11).

### Quality metrics

**Figure 7.**
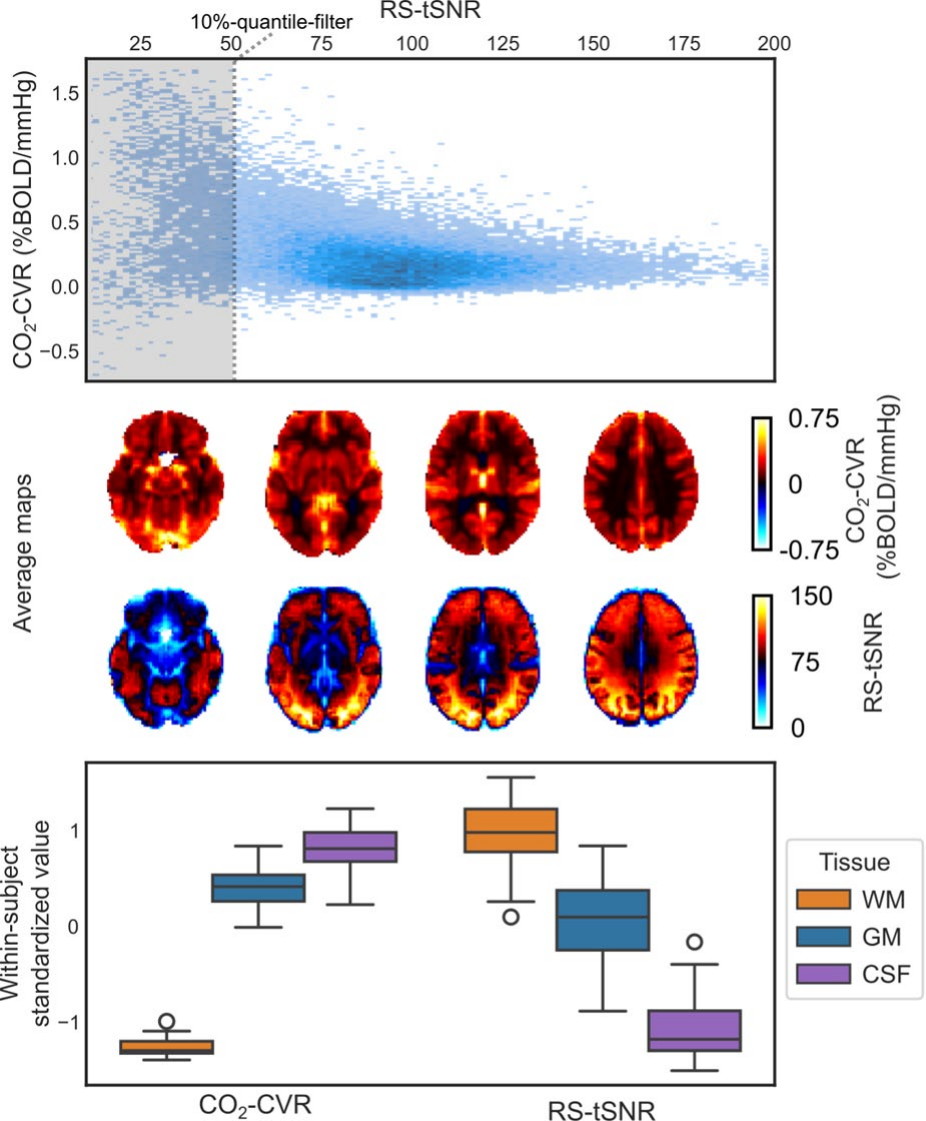
Comparison of tSNR and CVR. (top panel) Voxel-wise scatterplot of CO_2_-CVR versus RS-tSNR for a representative subject. The shaded area indicates the lowest 10% of tSNR values that would be excluded by the tSNR-quantile filter. (middle panel) Group-averaged maps of CO_2_-CVR and RS-tSNR, revealing spatial similarities, and (bottom panel) within-subject standardized values in WM, GM, and CSF, further illustrating tissue-level correspondence. Whiskers denote 1.5 times the interquartile range. WM: white matter; GM: gray matter; CSF: cerebrospinal fluid.

We found that identifying poor quality voxels using either *R*^2^, regressor *t*-value or fieldmap, had a moderate positive impact on repeatability, while tSNR had a negative effect (Supplementary Figure S5). This reduction appears to stem from tSNR preferentially removing voxels with high variability (Figure 7, top panel), which can attenuate correlation-based metrics that depend on overall variability within the dataset.

We also observe that both CVR and tSNR maps seem to highlight different tissue boundaries (Figure 7, middle panel) which is confirmed when comparing average values in various tissue types (Figure 7, bottom panel). In fact, all quality metrics, except fieldmaps, showed large tissue dependencies (Supplementary Figure S9).

Notably, the measures in Figure 7 were derived independently, CVR from the CO_2_ paradigm (CO_2_-CVR) and tSNR from the resting-state paradigm (RS-tSNR), yet similar associations were observed across other paradigm combinations, underscoring the robustness of this relationship.

## Discussion

In this study, we systematically evaluated how processing choices influence the repeatability of BOLD–CVR maps across common vascular paradigms. Our main findings were (i) applying temporal filtering and including motion-confounds can introduce substantial collinearity between the regressor and confounds, warranting caution when using them together and (ii) the temporal properties of the regressor impose fundamental limits on its ability to capture physiological delays, requiring care to avoid artifacts such as “fake steal.”

### Temporal filtering and motion-confounds create large collinearity

Our analysis of linear detrending, temporal filtering, and motion-confound reveals that processing steps in CVR analysis are not always additive. Specifically, when motion-confounds are included in the linear regression model, adding temporal filtering can be counterproductive, as filtering reduces degrees of freedom and increase collinearity between regressor and confounds (Figure 4, right column).

Without motion-confounds, temporal filtering showed little effect on test–retest repeatability if fundamental stimulus frequencies were preserved, for our CO_2_ paradigm this frequency was ~1/135 Hz (60 s CO_2_ + 75 s rest) and for BH ~1/60 Hz (15 s BH + 45 s rest) plus the first and second harmonics, supporting that CVR responses are primarily stimulus driven (Figure 4, left column). For RS, test–retest repeatability plateaued at 232.8 mHz (~1/4 s) and the correlation with CVR reference map showed a peak at approximately the same frequency (Supplementary Figure S8). This corresponds to typical respiratory cycles (~5–8 s), indicating that RS-CVR is primarily driven by spontaneous breathing. These results are consistent with Liu et al.,^9^ although they reported a peak at 116.4 mHz (~1/9 s), possibly reflecting small differences in breathing patterns between studies.

From the optimal perturbation analysis (Figure 4), we not that including motion-confounds tended to reduce test–retest repeatability for the CO_2_ and BH paradigms (only statistical significantly for CO_2_) and had little to no effect on RS paradigm. However, this likely reflects the generally low levels of head motion in our dataset. For the few subjects with a higher degree of head motion during BH (average FD > 0.2 mm), including motion-confounds improved repeatability (Supplementary Figure S4), suggesting that the relative trade-offs might be different when more motion is present. Caution is therefore needed before generalizing our results to other studies with greater and more variable head movement.

As a general recommendation, when motion-confounds are included in the linear regression model, additional processing steps that reduce the degrees of freedom, such as temporal filtering, should generally be avoided as this can increase the collinearity between the regressor and confounds. In contrast, when motion-confounds are omitted, temporal filtering is acceptable provided that fundamental stimulus frequencies, and relevant harmonics, are preserved.

### Temporal limitations of regressor can lead to fake steal

Anti-correlated voxels along the *x* = −*y* line in Figure 6 (first column, orange) likely reflect true CVR responses with inverted sign caused by regressor misalignment. Such sign inversions can mimic vascular steal, that is, “fake steal,” and arise when baseline segments of the regressor become aligned with plateau segments of the signal, and vice versa. Consistent with this interpretation, the test–retest delay differences for these anti-correlated voxels cluster around paradigm-specific times: ~60 s for CO_2_, 15 s for BH, and ~7 s for RS (Figure 6, fourth column). These values match the stimulus cycle lengths for CO_2_ and BH, and for RS they correspond to typical respiratory periods (~5–8 s). These voxels also show weaker overall correlation with the regressor (Figure 6, third column), which would make them more susceptible to misalignment.

Crucially, the consistent, paradigm-specific delays suggest that constraining the delay range, ideally within the first local minimum of the regressor’s autocorrelation (Figure 6, fifth column), can mitigate these fake steal artifacts. Since autocorrelation structures differ across paradigm, the safe delay window should be tailored accordingly. Indeed, such an approach is implemented by the advance package RapidTide.^44^ Another solution is to modify the autocorrelation structure itself, for instance by using an asymmetric stimulus. Alternatively, median filtering delay maps before CVR estimation (Supplementary Figure S11) also reduces anti-correlated voxels, suggesting that post hoc filtering can enhance estimate quality.

### Other important considerations in BOLD–CVR imaging

Below, we outline important considerations in BOLD–CVR imaging that are highlighted by our findings and align with broader knowledge in the field.

#### BOLD–CVR is influenced by baseline vascular factors

Our CVR–tSNR analysis shows that quality metric filtering must be applied cautiously, as aggressive filtering can bias results toward specific tissues or regions (Figure 7). Furthermore, the strong spatial correspondence between CVR and tSNR also suggests a shared underlying source.

This could be baseline vascular factors such as cerebral blood volume or deoxyhemoglobin concentration. Their influence is a known limitation of BOLD–CVR^45^ and it seems plausible that they be captured by fluctuation-based metrics like tSNR, especially since such fluctuations have previously been used to rescale fMRI signals.^46,47^ An alternative, not mutually exclusive, explanation is that both measures reflect true physiological CVR, consistent with tSNR being the inverse of resting-state fluctuation amplitude (RSFA), a known CVR proxy.^48^

Regardless of interpretation, the dependence of BOLD–CVR on baseline physiology means caution is warranted when comparing CVR values across regions or tissue types that may be modulated by varying baseline vascular factors.

#### Smoothing improves SNR but reduces subject and tissue contrasts

Our results support what is generally understood: spatial smoothing improves SNR but also decreases subject identifiability and increases tissue blending. This trade-off is evident comparing the within-subject and differential correlation, as well as the mean CVR values across tissue types as smoothing increases (Figure 3). (Note that the high average CVR observed in CSF arises because the CSF mask includes extracerebral regions that contain large venous sinuses.)

Choosing an optimal smoothing level therefore requires weighing SNR improvement against the need to preserve subject-specific and spatial detail. Larger kernels (e.g. 8 mm fwhm for 3 mm voxels at 3 T) may be appropriate for detecting broad CVR changes, such as those seen in steno-occlusive disease. However, for fine-grained effects, such as those relevant to small vessel disease, this degree of smoothing may obscure diagnostically important features.

Standard Gaussian smoothing also blends signals across tissue boundaries, complicating interpretation, especially considering the above discussion on baseline vascular factors and its influence on BOLD–CVR measurements. We therefore recommend alternative smoothing strategies that respect anatomical boundaries, such as tissue-specific or edge-preserving approaches (e.g. SUSAN in the FSL toolbox^49^), which offer a more appropriate balance between SNR improvement and spatial specificity.

#### Choice of regressor determines interpretation of CVR maps

Across all vascular paradigms, the global signal produced more repeatable CVR maps than the ET-CO_2_ regressor, even for the CO_2_ paradigm, where ET-CO_2_ itself showed high repeatability. This likely reflect the fact that ET-CO_2_ is only an indirect measurement of arterial CO_2_ and that a HRF links changes in blood gas concentrations to the resulting cerebral blood flow and BOLD responses. Much of this HRF is implicitly captured by the global signal, although it also incorporates non-CO_2_ contributions such as neural activity and regionally varying delays.^50^ Another limitation of using the global signal is that the resulting CVR estimates no longer retain physiologically interpretable units (e.g. %BOLD/mmHg).

An alternative explanation for the improved repeatability is that CVR may change between session. We observed a consistent decrease from test to retest, consistent with Sleight et al.,^51^ possibly reflecting reduced CO_2_ sensitivity after the initial exposure. Because the global signal scales with the overall BOLD response, using it as a regressor partially normalizes out such global shifts, yielding CVR maps that reflect relative rather than absolute reactivity.

This raises a conceptual question: should BOLD–CVR be interpreted as a quantitative physiological measure or as a spatial map of relative vascular responsiveness? And given BOLD’s dependence on baseline vascular factors, does a quantitative regressor truly make BOLD–CVR more quantitative?

In practice, the best regressor might depend on study goals. As both the global signal and ET-CO_2_ can be obtained with minimal effort, we recommend acquiring both and selecting whichever yields the most consistent and meaningful CVR estimates for the population and application of interest.

#### Lost signal can not be restored using SDC

Applying SDC results in little to no improvement in test–retest repeatability (Supplementary Figure S5). Perhaps expected given that the SDC is driven by stable anatomical features, specifically boundaries between tissue–air and tissue–bone. Our data also confirms the well-known dependence of distortion on boundary geometry and orientation relative to the *B*_0_ field, which determines both the direction and magnitude of field perturbations (Figure 2).^52^

Accordingly, the benefit of SDC in gradient-echo imaging lies in repositioning signal to its correct anatomical location, not restoring lost signal.^53^ This is illustrated in the bottom panel of Figure 2, where blue voxels in the anterior brain reflect apparent signal loss after SDC. Strong anterior field perturbations shift these voxels posteriorly into the brain, causing signal mixing that cannot be fully disentangled.

This discussion highlights the importance of planning BOLD–CVR experiments in advance to minimize signal loss. Adjusting the phase-encoding direction or tilting the acquisition slab can reduce dropout in specific regions, often, unfortunately, at the cost of increased distortion elsewhere.^54^

### Limitations

#### Single dataset

Our study examined processing parameters using a single dataset with a specific scanning protocol, which may limit the generalizability of some findings. For example, some toolboxes, such as AFNI, automatically adjust linear detrend order based on the scan length,^55^ why our results might not be transferable to studies with substantially different scan length than used in our study (5 min). Therefore, caution is warranted when applying our results to datasets acquired with different scanning protocols.

#### Only two measurements

Our repeatability assessment relied on just two measurements (test–retest). A more comprehensive evaluation would involve additional repetitions, though the optimal number is not straightforward to define. However, because our goal was not to estimate absolute repeatability with high precision but rather to identify trends and provide methodological insights, we consider two measurements sufficient for the purposes of this study.

#### Design parameters scope

Our analysis focused on a selected set of CVR processing parameters (Supplementary Table S1). While not exhaustive, these parameters span a broad cross-section of commonly used practices. Many alternative or more advanced approaches, such as ICA-based denoising, certainly exist, but we consider our chosen set to be broadly representative of current CVR processing strategies.

#### Vascular paradigm

This study compared several commonly used vascular paradigms in CVR imaging but was not exhaustive; for example, it did not include acetazolamide administration or vasoconstrictive challenges such as paced deep breathing. Although optimal processing strategies may differ between paradigms, we believe that many of the insights gained here remain broadly applicable.

#### Regressor

Although it is common in CVR imaging to convolve the physiological regressor with a HRF, there is no consensus on the most appropriate HRF, nor on whether the optimal HRF varies across vascular paradigms. Regional differences in venous drainage can further shape the HRF,^13^ which some approach by fitting voxel-wise HRFs.^14^ Given these complexities, we chose not to evaluate HRF convolution, as doing so would require dedicated methodological considerations beyond the scope of this work. We also note that using the global signal as a regressor can partially mimic the effect of HRF convolution, though it introduces its own limitations, as discussed above.

#### Repeatability metric

We assessed repeatability using correlation-based metrics computed across the whole-brain CVR maps and did not evaluate alternative approaches such as regional analyses or Bland–Altman comparisons. Although several metrics are available, correlation effectively captured both repeatability and reliability for our purposes. Because our aim was to provide practical insights into processing choices rather than an exhaustive comparison of repeatability metrics, we focused on a single, simple, and interpretable measure.

#### Emphasis on CVR amplitude

We focused our analysis on CVR amplitude and did not evaluate delay map repeatability or other dynamical aspects of the CVR response (e.g. response speed or shape). This decision allowed us to concentrate on the primary factors influencing CVR amplitude map generation without diluting the analysis with additional outcome measures.

#### CO_2_ sampling

Our setup estimated ET-CO_2_ without capturing full waveforms, limiting post hoc verification and potentially affecting generalizability, particularly during BH. However, in terms of repeatability of the regressor itself, the ET-CO_2_ timeseries showed similar performance as the global signal (Supplementary Figure S10), and the amount of missing ET-CO_2_ data during BH did not correlate with CVR map repeatability (Supplementary Material—CO_2_ sampling). Together, these observations indicate that our findings are reasonably robust despite the limitations of the CO_2_ sampling method.

## Conclusion

Our findings underscore the importance of standardization across processing strategies to improve reproducibility and facilitate comparison between studies. While this study focused on healthy subjects and widely used vascular paradigms, future work should explore how these findings translate to clinical populations and alternative stimulus protocols.

In offering this overview, we hope to contribute to a more transparent and accessible entry point into CVR imaging, while also encouraging greater consistency and comparability across studies.

## Supplemental Material

sj-docx-1-jcb-10.1177_0271678X261420026 – Supplemental material for How processing choices effect repeatability in BOLD–CVR imaging Supplemental material, sj-docx-1-jcb-10.1177_0271678X261420026 for How processing choices effect repeatability in BOLD–CVR imaging by Gustav Magnusson, Alex A Bhogal, Charalampos Georgiopoulos, Gunnar Cedersund, Lovisa Tobieson, Maria Engström and Anders Tisell in Journal of Cerebral Blood Flow & Metabolism
