# Supplementary material for: How processing choices effect repeatability in BOLD–CVR imaging
Source: J Cereb Blood Flow Metab. 2026 Apr 11:0271678X261420026. Online ahead of print. doi: 10.1177/0271678X261420026 (PMC13070108; doi:10.1177/0271678X261420026)
Supplement: sj-docx-1-jcb-10.1177_0271678X261420026 – Supplemental material for How processing choices effect repeatability in BOLD–CVR imaging [file sj-docx-1-jcb-10.1177_0271678X261420026.docx]

Supplementary material

[Design Parameters 2](#_Toc214813860)

[CO_2_ Sampling 3](#_Toc214813861)

[MRI Acquisition Parameters 6](#_Toc214813862)

[Discomfort 7](#_Toc214813863)

[Head Motion 9](#_Toc214813864)

[Univariate Sensitivity Analysis 10](#_Toc214813865)

[Linear Detrending, Temporal Filtering and Motion-Confounds Interaction 13](#_Toc214813866)

[Optimized Parameters 15](#_Toc214813867)

[Regressor versus CVR Map Repeatability 18](#_Toc214813868)

[Median Filtering of Delay Maps 20](#_Toc214813869)

[References 21](#_Toc214813870)

# Design Parameters

An overview of all design parameters considered is provided in Table S1, grouped into subcategories for clarity. Both the vascular paradigm and the various data processing strategies are treated as design parameters, although analyses were performed separately for each paradigm. For completeness, Table S1 also includes essential parameters of a typical CVR pipeline, such as the imaging protocol which has been studied elsewhere (1–4), although not specifically investigated here.

***Table S1.*** *Detailing the design parameters considered in this study, grouped into logical subcategories. A brief description and limitation made to each parameter is given, as well as investigated levels and the default value. For completeness we also include design parameters important for CVR imaging although not explicitly investigated here, such as stimulus and imaging protocol.*

|  | Design parameter | Description | Limitation | Investigated levels | Default |
| --- | --- | --- | --- | --- | --- |
| Acquisition | **Vascular paradigm** | The strategy used to create a vascular flow alternation to measure CVR | We considered inspired CO_2_, breath-hold (BH) and resting-state (RS) based CVR | CO_2_, BH or RS | CO_2_ |
|  | **Stimulus protocol** | The duration, strength and variation of the vascular stimulus | We only considered one protocol for each vascular paradigm, illustrated in Figure 1 in the main text | Not investigated | See Figure 1 in main text |
|  | **Stimulus sampling** | Technique to sample and measure the strength of the stimulus | We only considered sampled end-tidal CO_2_ using a facemask and a MR-compatible monitor | Not investigated | See Figure S1 and S2 |
|  | **Imaging protocol** | The method employed for measuring blood flow changes | We only considered BOLD-MRI imaging with parameters listed in Table S2 | Not investigated | See Table S2 |
| Pre-processing | **Image correction** | Including slice-timing- and motion-correction | We used the third-party program fMRIPrep (v.23.2.0a3) with default parameters | Not investigated | See main text |
|  | **Susceptibility distortion correction (SDC)** | Correcting for susceptibility induced distortion in BOLD images | We considered only dual-echo field-map based SDC, implemented in fMRIPrep | Binary: With or without SDC | With SDC |
|  | **Resampling to atlas space** | Registration and normalization of BOLD image to standard space | Using fMRIPrep, we considered only subject T1w space or standard MNI152NLin2009cAsym space | Binary: T1w or MNI | MNI |
|  | **Voxel-mask** | Strategy used for masking out voxels for analysis | We used Nilearn’s tool compute_epi_mask (EPI-mask) and fMRIPrep’s computed brain mask (brain-mask) | Binary: EPI-mask or brain-mask | EPI-mask |
| Post-processing | **Spatial smoothing** | Spatially smoothing the BOLD data to improve signal-to-noise ratio (SNR) | We used a gaussian kernel with variable full width at half maximum (fwhm) | fwhm = 0,2,3,4,5,6,7,8,9 mm | 5 mm |
|  | **Voxel- or parcel-based timeseries** | Conducting analysis on individual voxels or parcel aggregated timeseries | Parcel-based aggregation limited to the Desikan-Killiany atlas (aparc+aseg) | Binary: voxel- or parcel-based timeseries | Voxel |
|  | **Temporal upsampling** | Temporal upsample the data to improve regressor alignment | Only considered integer upsampling | Factor = 1,2,4  (1 = no upsampling) | 2 |
|  | **Linear detrending** | Remove linear trends in the data | We used polynomial basis functions with variable degree | Degree = 0,1,2,3  (0 = no detrending) | 1 |
|  | **Temporal filtering** | Temporal filtering of data | We used a Butterworth 6^th^ order low-pass filter with variable cutoff frequency | Cutoff frequency =  2^n^ x 7.275 mHz,  n ∈ {0,1,2…,6,∞} (n = ∞ => no filtering) | 116.4 mHz  (n = 4) |
| Regression | **Baseline measure** | Strategy to measure baseline and scale BOLD signals to percent signal change | We used either the overall-mean, mean during initial/final 15 s, interpolation between the initial/final 15 s. | Strategy: overall-mean, initial 15 s, final 15 s, interpolation | Overall-mean |
|  | **Regressor choice** | Choosing regressor to compute CVR metric | Either end-tidal CO_2_ (ET-CO_2_) trace of global BOLD signal | Binary: ET-CO_2_ or global timeseries | ET-CO_2_ for CO_2_/BH  global for RS |
|  | **Alignment bounds** | Align regressor to voxel BOLD timeseries within specific temporal bounds | Symmetrical bounds around 0 s (after regressor has been aligned to global BOLD signal) | Bounds = ± 0, 5, 8, 15, 30, 45 s, ∞ (± 0 s = no alignment; ∞ = no bounds) | ± 0 s |
|  | **Motion-confounds** | Inclusion of motion-confounds in linear regression model | Using 24 motion timeseries, excluding those with absolute regressor correlation above threshold | Threshold = 0, 0.25, 0.5, 0.75, 1 (0 = no confounds, 1 = all confounds) | 0 |
| Refinement | **Quality metric** | Identify and remove poor signal voxel using quality metrics | Using either R^2^, regressor t-value, fieldmap (fmap), or tSNR, thresholded to remove worst 10% of voxels | Quality metric = None, R^2^, t-value, fmap, tSNR | None |
|  | **Normalization** | Normalize CVR values to create relative measures | We normalized by either whole-brain or tissue specific CVR values, segmentation computed by FSL FAST | Normalize = None, WB, GM, WM, CSF | None |
|  | **Tissue of interest (TOI)** | Confine analysis to a specific tissue of interest | We used either whole-brain or tissue specific regions computed by FSL FAST | TOI = WB, GM, WM, CSF | WB |

# CO_2_ Sampling

Subjects’ CO₂ levels during CVR assessments was measured using an MRI patient monitoring system (Expression MR400, Philips Medical Systems, Orlando, USA), wirelessly connected to a secondary information portal (Expression IP5, Philips Medical Systems, Orlando, USA) from which data was exported. A key limitation of this setup is the inability to export the full CO₂ trace; only estimated end-tidal (ET) and inspired CO₂ values are available, sampled at a low temporal resolution (1 Hz). Consequently, we were unable to verify these estimates post hoc and had to rely on the device’s internal algorithm. In most cases, the algorithm appeared to perform reliably, as illustrated in the top-left panel of Figure S1, which displays raw MR400 output from an example subject during the CO₂ paradigm. However, the device occasionally failed to differentiate between ET and inspired CO₂, particularly during the CO₂-CVR experiment.


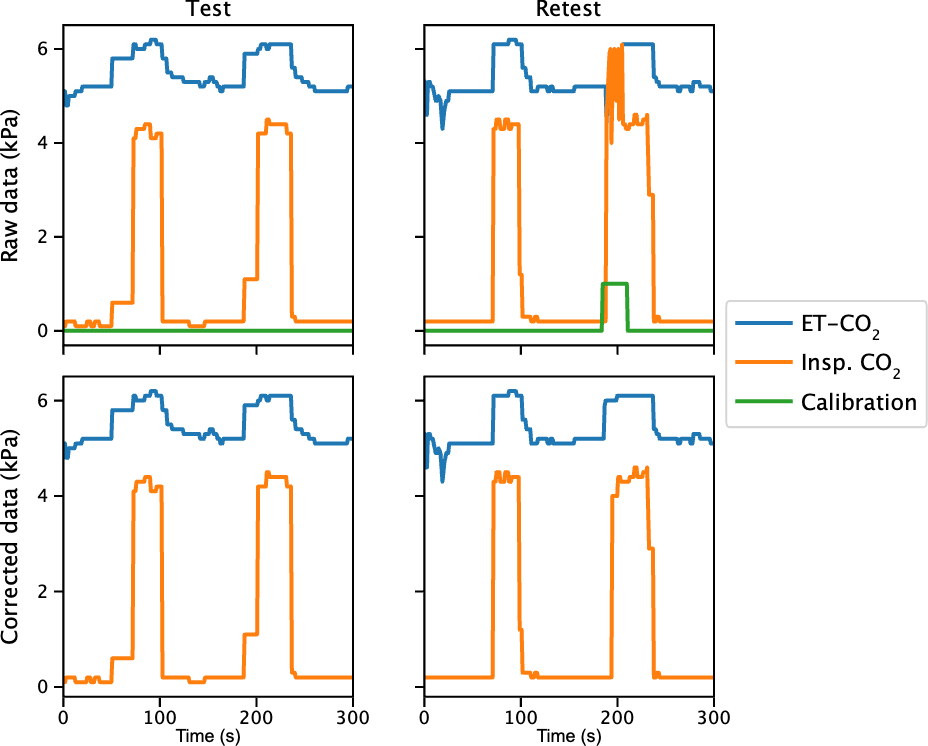

***Figure S1.*** *Example CO₂ data from a single subject during the CO₂ paradigm, shown for both the test and retest runs. Top panels: Raw ET-CO₂ and inspired CO₂ signals as recorded by the MR400 patient monitor, along with retrospectively identified calibration periods. Bottom panels: Corrected signals, in which ET-CO₂ and inspired CO₂ values during the calibration periods have been replaced with the local maximum and minimum values, respectively.*

During such “calibration” intervals, the MR400 substituted both ET and inspired CO₂ values with the raw CO₂ trace (still at 1 Hz resolution). These intervals could be identified retrospectively by detecting periods in which ET and inspired CO₂ values converged (i.e., difference < 1 kPa), as shown in the top-right panel of Figure S1. To correct these segments, we replaced the estimated ET and inspired CO₂ values with the local maximum and minimum, respectively, within a 12.5-second rolling window. The corrected data are illustrated in the bottom panels of Figure S1.

This correction successfully recovered the expected CO₂ pattern during the second inspired CO₂ block in the retest data. However, a comparison of the first and second blocks shows that the first block is substantially shorter. This discrepancy could stem from the MR400 failing to register the initial rise in CO₂, or it could reflect a genuinely shorter stimulus duration. Without access to the full CO₂ trace, it is difficult to determine the exact cause, highlighting a key limitation of our setup.

Despite this, the correction procedure appeared effective for the majority of subjects and runs in both the CO₂ and RS paradigms. In these cases, the resulting ET-CO₂ traces were deemed sufficiently reliable for use as regressors in the CVR analysis (after conversion from kPa to mmHg). The BH paradigm, however, introduced additional challenges due to missing data during the holds. Subjects were instructed to exhale immediately before and after each BH to facilitate interpolation. Nevertheless, because the MR400 only provided estimated values rather than the full CO₂ trace, post-BH ET-CO₂ values were occasionally missing or underestimated.


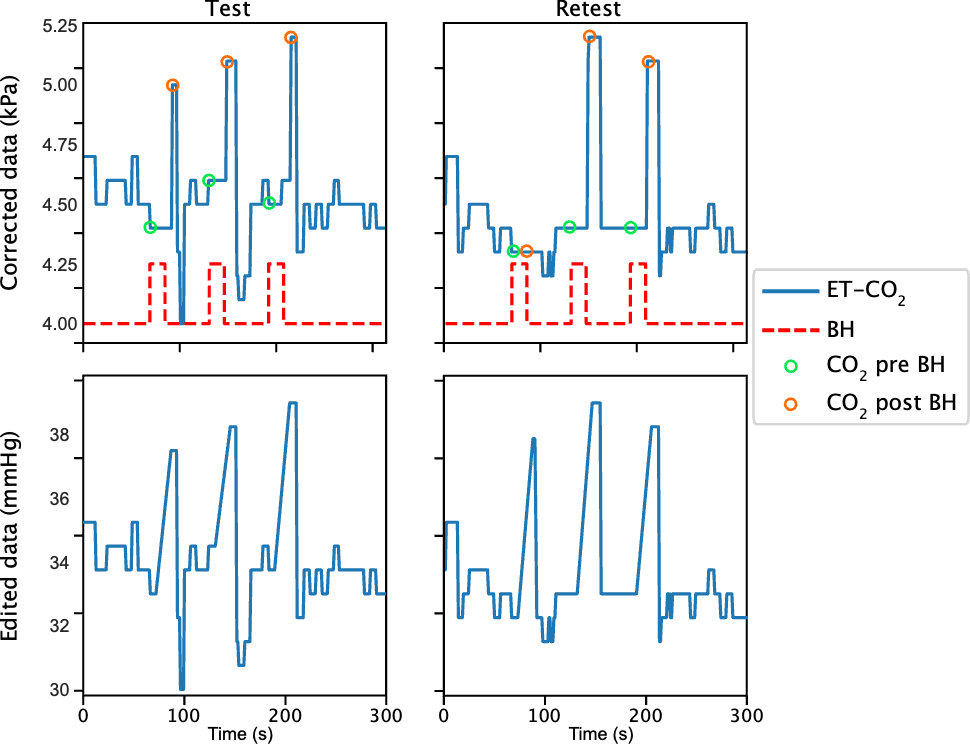

***Figure S2.*** *Additional processing of corrected BH ET-CO₂ data for a representative subject. In the test run (top-left), ET-CO₂ was successfully recorded before and after all BHs. The red dotted lines indicate the timing of each BH, with pre- and post-BH ET-CO₂ values marked in green and orange, respectively. In the retest run, however, the ET-CO₂ measurement following the first BH is missing. To address this, we replaced missing post-BH values by adding the mean ET-CO_2_ change of the remaining BHs to the pre-BH ET-CO_2_ value. The resulting edited data are shown in the bottom panels.*

Figure S2 (top row) shows corrected CO₂ data from a representative subject in the BH paradigm. In the test run, clear ET-CO₂ values were present before and after each BH, whereas the retest run lacked a post-BH measurement for the first hold. To address such cases, we quantified the relative ET-CO₂ increase for each of the three holds. Any increase smaller than 50% of the maximum observed increase was classified as an invalid post-BH measurement. Invalid values were then replaced by adding the mean increase from the remaining valid holds to the corresponding pre-BH ET-CO₂ value.

Visual inspection confirmed that every run had at least one reliable post-BH ET-CO₂ value, allowing us to retain all datasets. On average, 2.5 out of 3 holds per run were classified as valid. Importantly, subjects with fewer valid measurements did not show lower CVR map repeatability than those with more valid measurements, indicating that the correction procedure did not materially affect the results.

# MRI Acquisition Parameters

***Table S2:*** *MRI acquisition parameters for the sequences used in the study.*

| Sequence | Flip angle (◦) | TR (s) | TE (ms) | Voxel-size (mm) | Matrix | Sequence specific parameters |
| --- | --- | --- | --- | --- | --- | --- |
| T1w MPRAGE | 8 | 2.3 | 2.36 | 0.9 x 0.87 x 0.87 | 208 x 288 x 288 | TI: 0.9s; GRAPPA: 3; ref. lines: 24 |
| GRE-fieldmap | 60 | 0.52 | 4.92/7.38 | 3 x 3 x 3 | 68 x 68 x 45 |  |
| GRE-EPI BOLD | 56 | 0.878 | 24 | 3 x 3 x 3 | 68 x 68 x 45 | MB-factor 3; #meas: 342; phase-encoding: AP |

# Discomfort

After the CVR experiment, subjects were asked to quantify their discomfort from the different vascular paradigms. We clarified what was unique to each specific paradigm to help them separate out other sources of discomfort: breathing in CO₂ during the CO₂-CVR exam, holding their breath and following the instructions on the screen for the BH-CVR exam, fixating on the cross during the RS-CVR exam. Further, we asked them to rate the discomfort arising from sources common to all paradigms: wearing a facemask and laying in the MRI-scanner. They graded their level of discomfort on a scale going from: No, Little, Moderate, Strong, Extreme discomfort. Given the more invasive nature of CO₂ inhalation, we specifically asked if the subject noticed the CO₂ and if they experience any of the following symptoms during the CO₂ paradigm: shortness of breath, dry mouth, dizziness, headache, tingling sensation, anxiety, sensation of increased heart rate. The subjects were also allowed to leave any additional comment regarding their experience of the experiment.

We found no significant difference in level of experienced discomfort between vascular paradigms (Friedmann’s test; p = 0.17), see Figure S3. However, if we included discomfort from facemask and MRI-scanner, we found significant differences (Friedmann’s test; p = 0.01) and the pairwise analysis showed significant difference between BH and facemask (Wilcoxon signed rank-test; p = 0.02; Bonferroni corrected).


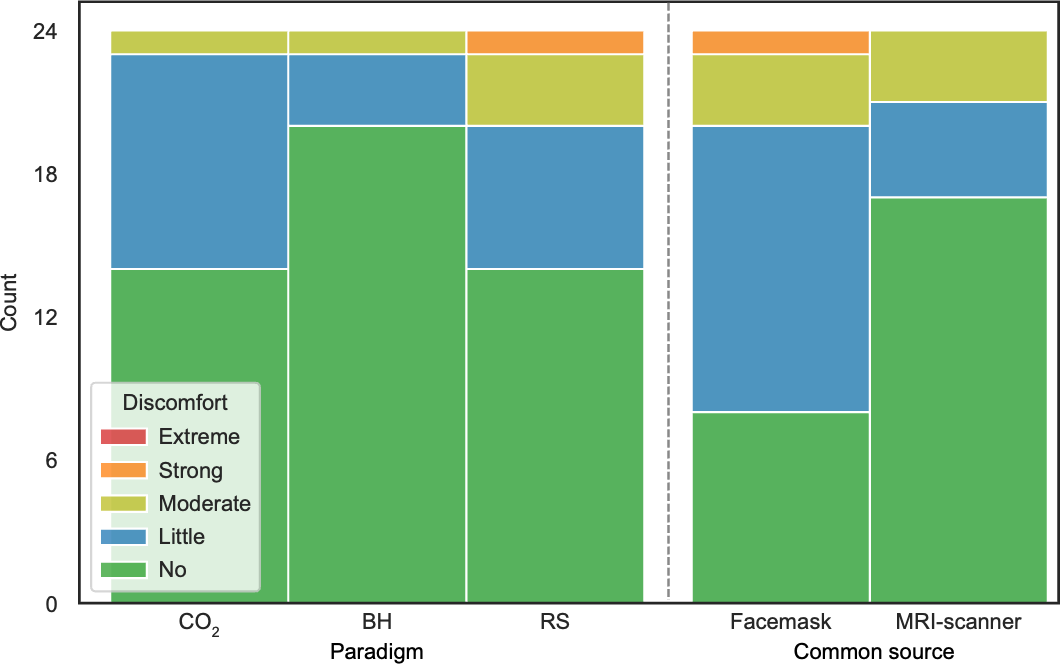


***Figure S3.*** *Self-reported experienced discomfort from vascular paradigms: CO_2_, BH and RS, as well as from common sources: wearing the facemask and being the MRI-scanner.*

Comparing in detail the reported level of discomfort from the three vascular paradigms, we see that RS tended to have higher levels than CO_2_ and BH, with four subjects experiencing moderate to strong discomfort, compared to only one subject reporting moderate discomfort during CO_2_ and BH. Looking at the optional comment subjects could leave; it seems that the higher level of discomforts experienced during RS arose from having to fixate on the cross on the bright projector screen during the exam which caused irritations to their eyes. The idea of having the subjects fixating on a cross during RS was mainly for them to stay awake and we had not anticipated that this would cause any discomfort. This part of the RS paradigm could easily be adapted by either reducing the brightness of the projector or removing the fixation on the cross completely.

We conclude that that none of the vascular paradigms caused any great discomfort for the majority of subjects and that the source of discomfort is not always what you expect, for example the brightness of the projector screen during RS. However, we investigated a young and motivated group (given that they volunteered for the study) and the level of discomfort reported here is therefore likely to be biased towards lower values compared to the population average.

For the CO_2_ paradigm, a majority of subjects did notice the CO_2_ (63%, n = 15) and the most common symptoms were dry mouth (38%, n = 9) and shortness of breath (25%, n = 6) (Table S3). Three subject reported tingling sensation (13%), two dizziness (8%) and 1 anxiety (4%) . For those subjects who did not notice the CO_2_, two reported no symptoms and five reported dry mouth as the only symptom.

***Table S3:*** *Questions and answers specific to the CO_2_ paradigm.*

| Questions and answers | Number of subjects (percentage) |
| --- | --- |
| *Did you notice the CO_2_?* |  |
| Yes | 15 (63%) |
| No | 7 (29%) |
| Maybe | 2 (8%) |
|  |  |
| *Symptoms relating to inspired CO_2_* |  |
| Dry mouth | 9 (38%) |
| Shortness of breath | 6 (25%) |
| Tingling sensation | 3 (13%) |
| Dizziness | 2 (8%) |
| Anxiety | 1 (4%) |
| Headache | 0 (0%) |
| Sensation of increased heart rate | 0 (0%) |

An easy solution to the problem of dry mouth could be to include a passive heat and moisture exchange (HME) filter, which we did not.

# Head Motion

Head motion was quantified using mean framewise displacement (FD) from FSL MCFLIRT (via fMRIPrep), averaged across runs. Pairwise comparisons using paired t-tests with Holm correction showed that average FD was significantly higher in BH (0.14 mm) than RS (0.11 mm; p = 0.04) but did not differ significantly between BH and CO₂ (0.12 mm) or between CO₂ and RS.

Increase in test-retest ICC(C,1) after including motion-confounds in the linear regression model, did not correlate significantly with average FD during either the CO_2_ or RS paradigm (Figure S4). This can in part be explained by the fact that subjects overall displayed very little motion during these paradigms. For BH on the other hand, a few subjects displayed higher degree of head motion (average FD > 0.2 mm) where including motion-confounds resulted in improved repeatability and an overall significant correlation (r = 0.74, p < 0.001; using Holm correction for multiple comparison).


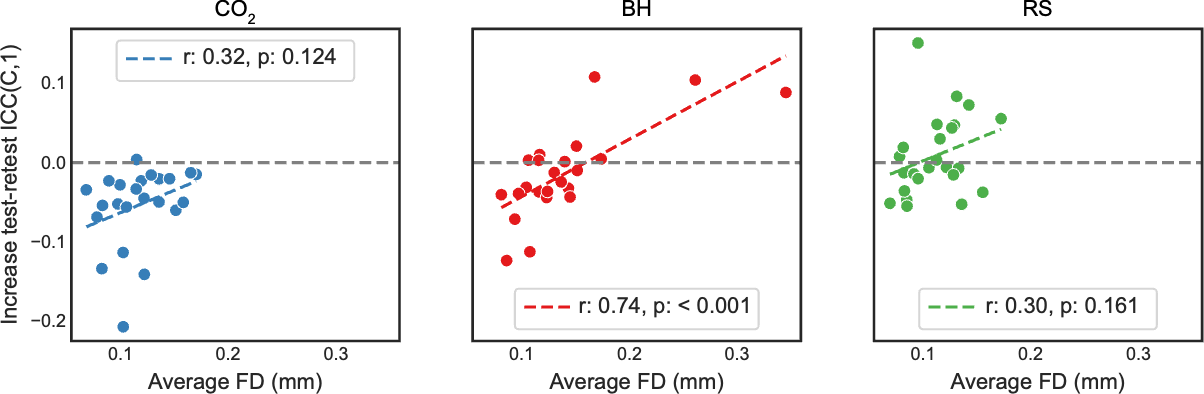


***Figure S4.*** *Improvement in repeatability, expressed as the increase in test–retest ICC(C,1) after including motion confounds in the linear regression model, plotted against head motion (average framewise displacement, FD) across vascular paradigms.* *For each paradigm, the correlation coefficient r and corresponding p-value are reported. For the CO₂ and RS paradigms, improvements in ICC(C,1) show little relationship with FD, likely because subjects overall exhibited low amount of motion. In contrast, in the BH paradigm, two subjects showed substantially higher amount of motion (average FD > 0.2 mm), and both benefited from the inclusion of motion-confounds, yielding a significant overall correlation.*

Note that test-retest ICC(C,1) improvements were computed using paradigm-specific optimal parameters for linear detrending order and low-pass cut-off frequency, which differed whether motion-confounds were included or not.

# Univariate Sensitivity Analysis


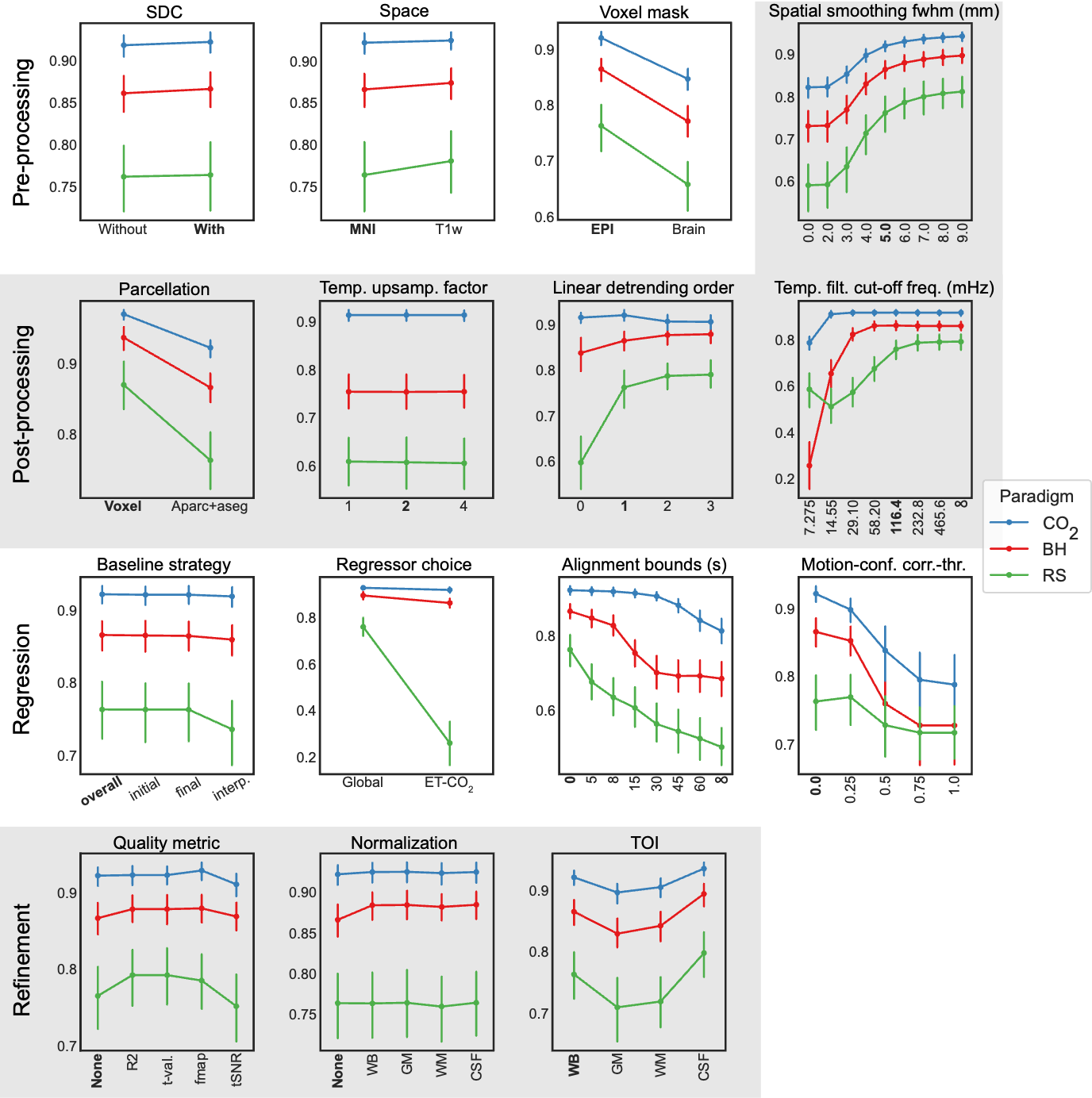


***Figure S5.*** *Result from initial univariate sensitivity analysis, repeatability between test-retest (measured using ICC(C,1)) was computed as each design parameter was univariately perturbated while the others were kept at their default value (marked with bold text). The only exception being alignment bounds set to 15 s when changing temporal upsampling factor to see an effect, and regressor choice which default value depended on vascular paradigm (ET-CO_2_ for CO_2_/BH and global signal for RS). The analysis was done across the three different vascular paradigms: CO_2_, BH and RS and the parameters have been grouped into four categories: pre/post-processing, regression and refinement. See Table S1 for more detail of each parameter.*

***Table S4.*** *Results from univariate analysis, using AnovaRM to test for significance. Shown for each vascular paradigm and factor: Uncorrected F-values and degrees of freedom for numerator and denominator (df^n^, df^d^), Huynh-Feldt correction factor (HF), corresponding p-value and the partial effect size (η^2^). Uncorrected p-values are marked with HF = Udef. (correction undefined; factor with two levels) or HF = Sing. (singular covariance matrix; unable to compute correction factor).*

|  |  | CO_2_ | | | | | | | | | BH | | | | | | | | | RS | | | | | | |
| --- | --- | --- | --- | --- | --- | --- | --- | --- | --- | --- | --- | --- | --- | --- | --- | --- | --- | --- | --- | --- | --- | --- | --- | --- | --- | --- |
|  | *Factor* | *F(df^n^, df^d^)* | | *HF* | *p* | | *η^2^* | | *F(df^n^, df^d^)* | | | *HF* | | *p* | | *η^2^* | | *F(df^n^, df^d^)* | | | *HF* | | *p* | | *η^2^* | |
| Preproc. | SDC | *F(1, 23): 4.1* | *Udef.* | | | *0.053* | | *0.15* | | *F(1, 23): 4.4* | | | *Udef.* | | *0.048* | | *0.16* | | *F(1, 23): 1.5* | | | *Udef.* | | *0.23* | | *0.06* |
|  | Space | *F(1, 23): 2.1* | *Udef.* | | | *0.16* | | *0.08* | | *F(1, 23): 39.7* | | | *Udef.* | | *< 0.001* | | *0.63* | | *F(1, 23): 40.7* | | | *Udef.* | | *< 0.001* | | *0.64* |
|  | Voxel mask | *F(1, 23): 444.3* | *Udef.* | | | *< 0.001* | | *0.95* | | *F(1, 23): 230.7* | | | *Udef.* | | *< 0.001* | | *0.91* | | *F(1, 23): 225.7* | | | *Udef.* | | *< 0.001* | | *0.91* |
| Postproc. | Spatial smoothing | *F(8, 184): 703.8* | *0.13* | | | *< 0.001* | | *0.97* | | *F(8, 184): 246.9* | | | *0.13* | | *< 0.001* | | *0.91* | | *F(8, 184): 237.7* | | | *0.13* | | *< 0.001* | | *0.91* |
|  | Parcellation | *F(1, 23): 375.3* | *Udef.* | | | *< 0.001* | | *0.94* | | *F(1, 23): 147.4* | | | *Udef.* | | *< 0.001* | | *0.87* | | *F(1, 23): 128.4* | | | *Udef.* | | *< 0.001* | | *0.85* |
|  | Temporal upsampling | *F(2, 46): 0.8* | *0.91* | | | *0.44* | | *0.03* | | *F(2, 46): 1.3* | | | *0.69* | | *0.28* | | *0.05* | | *F(2, 46): 27.5* | | | *0.75* | | *< 0.001* | | *0.54* |
|  | Linear detrending | *F(3, 69): 11.8* | *0.57* | | | *< 0.001* | | *0.34* | | *F(3, 69): 13.1* | | | *0.57* | | *< 0.001* | | *0.36* | | *F(3, 69): 48.8* | | | *0.44* | | *< 0.001* | | *0.68* |
|  | Temporal filtering | *F(7, 161): 189.5* | *0.19* | | | *< 0.001* | | *0.89* | | *F(7, 161): 138.1* | | | *0.26* | | *< 0.001* | | *0.86* | | *F(7, 161): 44.5* | | | *0.35* | | *< 0.001* | | *0.66* |
| Regression | Baseline strategy | *F(3, 69): 8.8* | *0.39* | | | *0.0043* | | *0.28* | | *F(3, 69): 9.0* | | | *0.43* | | *0.0029* | | *0.28* | | *F(3, 69): 24.1* | | | *0.34* | | *< 0.001* | | *0.51* |
|  | Regressor choice | *F(1, 23): 14.8* | *Udef.* | | | *< 0.001* | | *0.39* | | *F(1, 23): 24.1* | | | *Udef.* | | *< 0.001* | | *0.51* | | *F(1, 23): 131.1* | | | *Udef.* | | *< 0.001* | | *0.85* |
|  | Alignment bounds | *F(7, 161): 109.3* | *0.20* | | | *< 0.001* | | *0.83* | | *F(7, 161): 164.5* | | | *0.23* | | *< 0.001* | | *0.88* | | *F(7, 161): 140.7* | | | *0.39* | | *< 0.001* | | *0.86* |
|  | Motion-confounds | *F(4, 92): 69.6* | *0.58* | | | *< 0.001* | | *0.75* | | *F(4, 92): 24.9* | | | *Sing.* | | *< 0.001* | | *0.52* | | *F(4, 92): 8.0* | | | *Sing.* | | *< 0.001* | | *0.26* |
| Refinement | Quality metric | *F(4, 92): 77.7* | *Sing.* | | | *< 0.001* | | *0.77* | | *F(4, 69): 13.0* | | | *Sing.* | | *< 0.001* | | *0.36* | | *F(3, 69): 36.6* | | | *Sing.* | | *< 0.001* | | *0.61* |
|  | Normalization | *F(4, 92): 5.1* | *0.29* | | | *0.029* | | *0.18* | | *F(4, 92): 9.1* | | | *0.26* | | *0.0054* | | *0.28* | | *F(4, 92): 13.7* | | | *0.39* | | *< 0.001* | | *0.37* |
|  | TOI | *F(3, 69): 100.4* | *0.77* | | | *< 0.001* | | *0.81* | | *F(3, 69): 109.9* | | | *0.68* | | *< 0.001* | | *0.83* | | *F(3, 69): 62.3* | | | *0.78* | | *< 0.001* | | *0.73* |

*Pre-processing*

Among the pre-processing design parameters, none appear to exert a strong influence on repeatability, with the possible exception of the voxel mask (Figure S5). As illustrated in Figure S6, the average voxel-mask varies noticeably across conditions. When susceptibility distortion correction (SDC) is applied, the resulting brain mask includes a substantial number of voxels outside the brain. Interestingly, this effect is absent when SDC is not used, compare the top-right and bottom-right masks in the figure. One possible explanation is that fMRIPrep adapts the brain mask to encompass regions with significant field distortions during SDC, potentially in an effort to preserve signal in those areas.


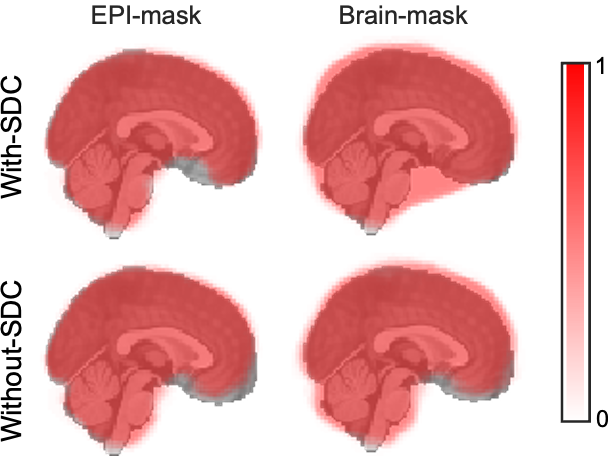


***Figure S6****. Average mask using either NiLearn compute_epi_mask (EPI-mask) or brain mask from the outputs of fMRIPrep (brain-mask), with and without susceptibility distortion correction (SDC).*

*Post-processing*

All three vascular paradigms exhibit similar trends across most post-processing parameters. Spatial smoothing leads to a marked initial improvement in repeatability, followed by a plateau (Figure S5). Using parcellation (aparc+aseg) instead of voxel-wise time series yields a substantial increase in repeatability across paradigms. In contrast, temporal upsampling, applied with alignment bounds set to 15 s, has minimal to no effect.

However, differences emerge for linear detrending and temporal filtering. For the RS paradigm, repeatability improves notably with the addition of detrending. In contrast, for the CO₂ paradigm, repeatability slightly declines as the detrending order increases beyond 1. With temporal filtering, all paradigms eventually reach a plateau in repeatability as the low-pass cutoff frequency increases, but the specific frequency at which this occurs varies by paradigm.

*Regression*

The choice of baseline estimation strategy, using either the overall mean, the initial 15 s mean, or the final 15 s mean, had little effect, while linear interpolation between initial and final means seemed to reduce repeatability across paradigms (Figure S5). In contrast, regressor selection shows a disproportionate effect on the RS paradigm compared to CO₂ and BH.

Allowing for regressor alignment by increasing the alignment bounds generally had a negative effect on repeatability. However, this effect was paradigm-specific: for CO₂, repeatability declined only with large bounds (>30 s), whereas for RS, the decline occurred even at small bounds.

Finally, the motion-confound correlation threshold negatively impacted repeatability across all paradigms.

*Refinement*

For quality metric filtering, we note that removing outliers identified using tSNR reduced repeatability, while the other wuality metrics marginally improved repeatability (Figure S5) . Normalization by different tissue doesn’t have a large effect repeatability. For tissue of interest (TOI), we note that GM has the lowest repeatability while CSF the highest, this probably reflect the different magnitude and variability of CVR values seen in these regions (Figure 7, main text)

# Linear Detrending, Temporal Filtering and Motion-Confounds Interaction

***Table S5.*** *Results from interaction analysis between linear detrending, temporal filtering and motion-confounds, using AnovaRM to test for significance. Shown for each vascular paradigm and factor: Uncorrected F-values and degrees of freedom for numerator and denominator (df^n^, df^d^), Huynh-Feldt correction factor (HF), corresponding p-value and the partial effect size (η^2^). Uncorrected p-values are marked with HF = Sing. (singular covariance matrix; unable to compute correction factor).*

|  | CO_2_ | | | | | | | BH | | | | | | | | | RS | | | | | | | | |
| --- | --- | --- | --- | --- | --- | --- | --- | --- | --- | --- | --- | --- | --- | --- | --- | --- | --- | --- | --- | --- | --- | --- | --- | --- | --- |
| Factor | *F(df^n^, df^d^)* | | *HF* | *p* | | *η^2^* | | | *F(df^n^, df^d^)* | | *HF* | | *p* | | *η^2^* | | | *F(df^n^, df^d^)* | | *HF* | | *p* | | *η^2^* | |
| Linear detrending | F(3, 69): 3.9 | 0.64 | | | 0.030 | | 0.14 | | | F(3, 69): 2.3 | | 0.75 | | 0.11 | | 0.09 | | | F(3, 69): 26.6 | | 0.49 | | < 0.001 | | 0.54 |
| Temporal filtering | F(7, 161): 554.8 | 0.30 | | | < 0.001 | | 0.96 | | | F(7, 161): 473.1 | | 0.38 | | < 0.001 | | 0.95 | | | F(7, 161): 346.8 | | 0.32 | | < 0.001 | | 0.94 |
| Motion-confounds | F(4, 92): 402.2 | 0.60 | | | < 0.001 | | 0.95 | | | F(4, 92): 109.1 | | 0.38 | | < 0.001 | | 0.83 | | | F(4, 92): 43.2 | | 0.41 | | < 0.001 | | 0.65 |
| Lin. detr. : temp. filt. | F(21, 483): 5.6 | 0.46 | | | < 0.001 | | 0.20 | | | F(21, 483): 1.7 | | 0.43 | | 0.097 | | 0.07 | | | F(21, 483): 1.7 | | 0.27 | | 0.13 | | 0.07 |
| Lin, detr. : mot.-conf. | F(12, 276): 6.6 | 0.54 | | | < 0.001 | | 0.22 | | | F(12, 276): 7.8 | | 0.52 | | < 0.001 | | 0.25 | | | F(12, 276): 25.9 | | 0.43 | | < 0.001 | | 0.53 |
| Mot.-conf. : temp. filt. | F(28, 644): 107.0 | Sing. | | | < 0.001 | | 0.82 | | | F(28, 644): 61.1 | | Sing. | | < 0.001 | | 0.73 | | | F(28, 644): 45.1 | | Sing. | | < 0.001 | | 0.66 |
| Lin. detr. : temp. filt. : mot.-conf. | F(84, 1932): 4.2 | Sing. | | | < 0.001 | | 0.16 | | | F(84, 1932): 2.1 | | Sing. | | < 0.001 | | 0.08 | | | F(84, 1932): 1.8 | | Sing. | | < 0.001 | | 0.07 |


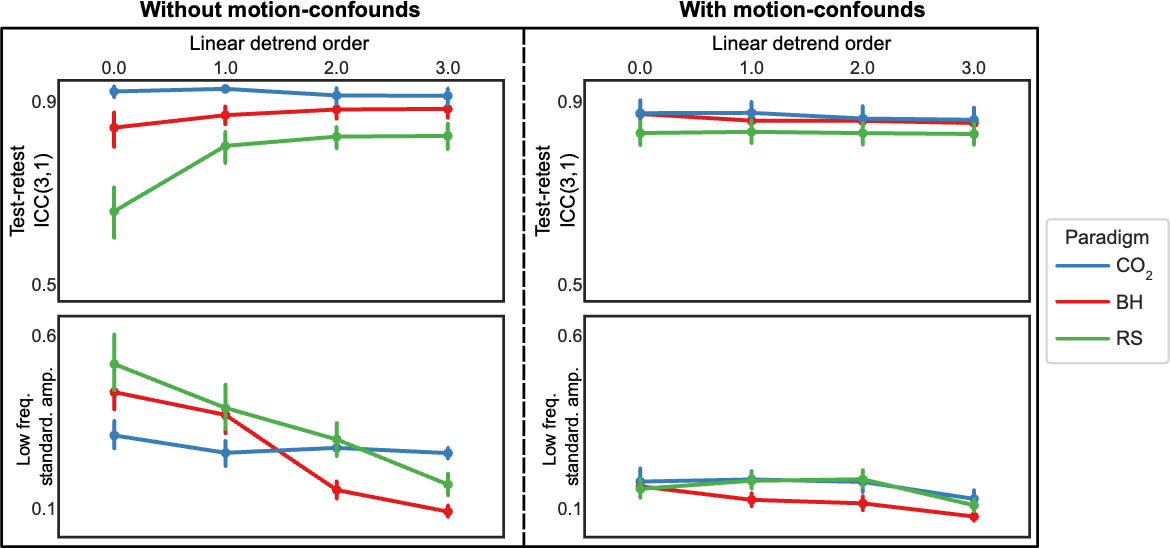


***Figure S7.*** *Interaction between linear detrending and motion-confounds. In the absence of motion-confounds, increasing the linear detrend order results in a positive trend in repeatability for both BH and RS paradigms. However, this effect is eliminated when motion-confounds are included. One possible explanation is that, without motion-confounds, linear detrending removes low-frequency components, visible in the lower left panel. When motion-confounds are included, these low-frequency components appear to have already been removed, compare with the lower right panel. Here we used no temporal filtering (infinite low-pass cutoff frequency) given the interaction with motion-confounds (see Figure 4 in main text). Low frequency refers to the lowest frequency in the FFT spectrum, which for our timeseries was 3.5 mHz (~1/285 s). The spectra were calculated on standardized signals.*

## Optimized Parameters

***Table S6.*** *Results from optimized perturbation analysis for linear detrending, temporal filtering and motion-confounds. Each parameter is perturbated while the other two are fixed to their empirical optimal value in terms of test-retest ICC(C,1). An AnovaRM model was used to test for significance. Shown for each vascular paradigm and factor: Uncorrected F-values and degrees of freedom for numerator and denominator (df^n^, df^d^), Huynh-Feldt correction factor (HF), corresponding p-value and the partial effect size (η^2^). Uncorrected p-values are marked with HF = Sing. (singular covariance matrix; unable to compute correction factor).*

|  | CO_2_ | | | | | | | | | BH | | | | | | | | RS | | | | | | | |
| --- | --- | --- | --- | --- | --- | --- | --- | --- | --- | --- | --- | --- | --- | --- | --- | --- | --- | --- | --- | --- | --- | --- | --- | --- | --- |
| Factor | *F(df^n^, df^d^)* | *HF* | | *p* | | *η^2^* | | *F(df^n^, df^d^)* | | | *HF* | | *p* | | *η^2^* | | *F(df^n^, df^d^)* | | | *HF* | | *p* | | *η^2^* | |
| Linear detrending | F(3, 69): 8.9 | | 0.52 | | 0.0016 | | 0.28 | | F(3, 69): 2.0 | | | 0.48 | | 0.16 | | 0.08 | | | F(3, 69): 0.9 | | 0.54 | | 0.40 | | 0.04 |
| Temporal filtering | F(7, 161): 125.8 | | 0.18 | | < 0.001 | | 0.85 | | F(7, 161): 143.2 | | | 0.25 | | < 0.001 | | 0.86 | | | F(7, 161): 44.6 | | 0.41 | | < 0.001 | | 0.66 |
| Motion-confounds | F(4, 92): 48.8 | | Sing. | | < 0.001 | | 0.68 | | F(4, 92): 1.7 | | | Sing. | | 0.15 | | 0.07 | | | F(4, 92): 0.5 | | Sing. | | 0.74 | | 0.02 |

*
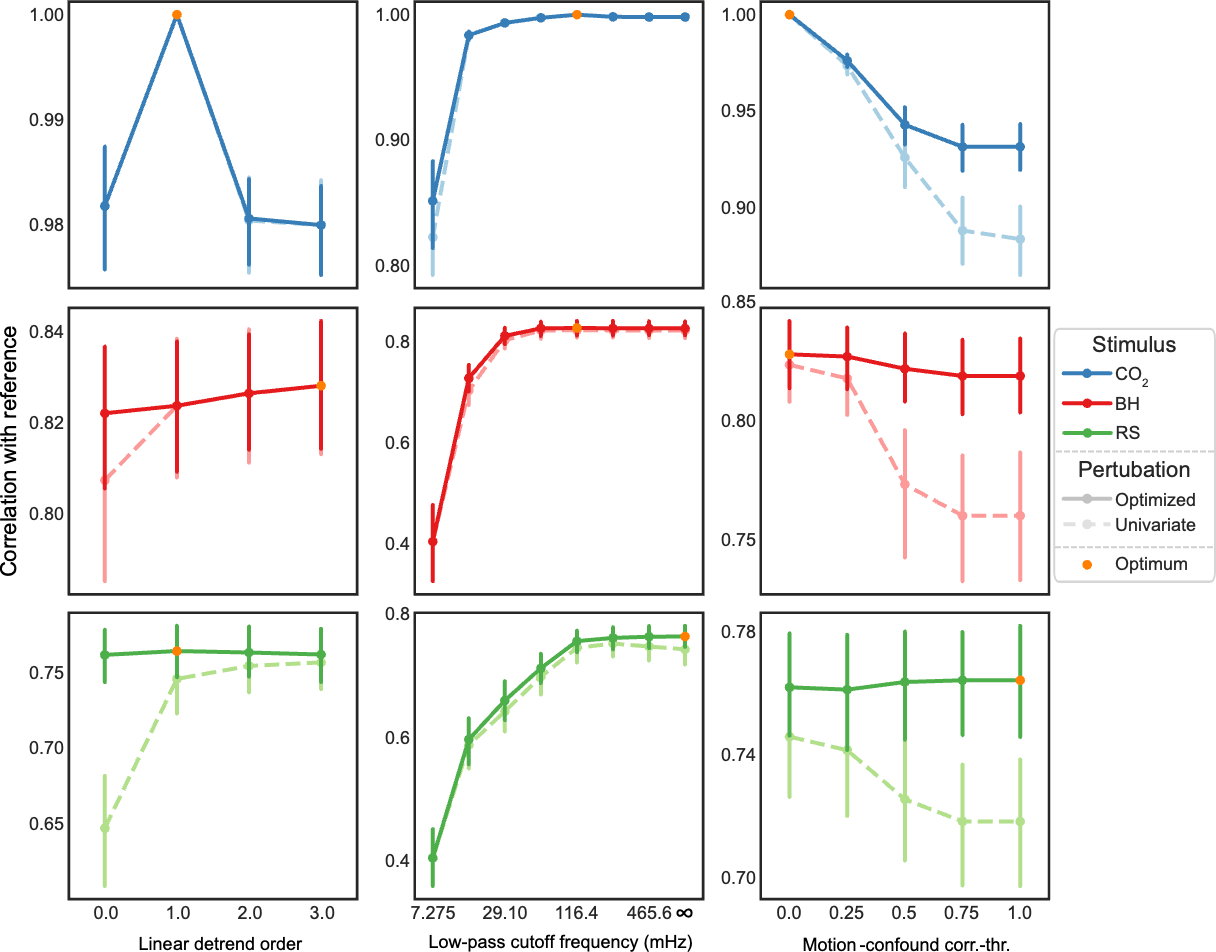
*

***Figure S8.*** *Correlation with reference using optimized and univariate perturbation. Repetition of the repeatability analysis, however now using whole-brain Pearson correlation with reference, the CO_2_ paradigm with optimum parameters (see top row), instead of test-retest ICC(C,1). For optimized perturbation, each parameter is perturbated while the other two are fixed to their empirical optimal value, in terms for correlation with reference. For univariate perturbation, each parameter is perturbated while the other two are held at their default value (see Table S1).*

***Table S7.*** *Results from the repeated optimized perturbation analysis,* *now using whole-brain Pearson correlation with reference, the CO_2_ paradigm with optimum parameters (see top row of Figure S8), instead of test-retest ICC(C,1). Each parameter is perturbated while the other two are fixed to their empirical optimal value in terms of correlation with reference. An AnovaRM model was used to test for significance. Shown for each vascular paradigm and factor: Uncorrected F-values and degrees of freedom for numerator and denominator (df^n^, df^d^), Huynh-Feldt correction factor (HF), corresponding p-value and the partial effect size (η^2^). Uncorrected p-values are marked HF = Sing. (singular covariance matrix; unable to compute correction factor).*

|  | CO_2_ | | | | | | | | | BH | | | | | | | | RS | | | | | | | |
| --- | --- | --- | --- | --- | --- | --- | --- | --- | --- | --- | --- | --- | --- | --- | --- | --- | --- | --- | --- | --- | --- | --- | --- | --- | --- |
| Factor | *F(df^n^, df^d^)* | *HF* | | *p* | | *η^2^* | | *F(df^n^, df^d^)* | | | *HF* | | *p* | | *η^2^* | | *F(df^n^, df^d^)* | | | *HF* | | *p* | | *η^2^* | |
| Linear detrending | *F(3, 69): 10911.6* | | *0.42* | | *< 0.001* | | *1.00* | | *F(3, 69): 0.8* | | | *0.42* | | *0.40* | | *0.03* | | | *F(3, 69): 0.1* | | *0.39* | | *0.83* | | *0.00* |
| Temporal filtering | *F(7, 161): 10630.9* | | *0.51* | | *< 0.001* | | *1.00* | | *F(7, 161): 136.9* | | | *0.21* | | *< 0.001* | | *0.86* | | | *F(7, 161): 136.5* | | *0.39* | | *< 0.001* | | *0.86* |
| Motion-confounds | *F(4, 92): 50481.9* | | *Sing.* | | *< 0.001* | | *1.00* | | *F(4, 92): 1.2* | | | *Sing.* | | *0.31* | | *0.05* | | | *F(4, 92): 0.1* | | *Sing.* | | *0.99* | | *0.00* |

***Table S8.*** *Results from the repeated univariate perturbation analysis, now using whole-brain Pearson correlation with reference, the CO_2_ paradigm with optimum parameters (see top row of Figure S8), instead of test-retest ICC(C,1). Each parameter is perturbated while the other two are held at their default value, see Table S1. An AnovaRM model was used to test for significance. Shown for each vascular paradigm and factor: Uncorrected F-values and degrees of freedom for numerator and denominator (df^n^, df^d^), Huynh-Feldt correction factor (HF), corresponding p-value and the partial effect size (η^2^). Uncorrected p-values are marked with HF = Sing. (singular covariance matrix; unable to compute correction factor).*

|  | CO_2_ | | | | | | | | BH | | | | | | | RS | | | | | | | | |
| --- | --- | --- | --- | --- | --- | --- | --- | --- | --- | --- | --- | --- | --- | --- | --- | --- | --- | --- | --- | --- | --- | --- | --- | --- |
| Factor | *F(df^n^, df^d^)* | |  | *p* | | *η^2^* | *F(df^n^, df^d^)* | | |  | | *p* | | | *η^2^* | | *F(df^n^, df^d^)* | | |  | | *p* | | *η^2^* |
| Linear detrending | *F(3, 69): 10784.0* | *0.42* | | | *< 0.001* | *1.00* | | *F(3, 69): 5.4* | | | *0.40* | | *0.023* | *0.19* | | | | *F(3, 69): 39.8* | *0.41* | | *< 0.001* | | *0.63* | |
| Temporal filtering | *F(7, 161): 10862.6* | *0.50* | | | *< 0.001* | *1.00* | | *F(7, 161): 134.7* | | | *0.21* | | *< 0.001* | *0.85* | | | | *F(7, 161): 143.4* | *0.36* | | *< 0.001* | | *0.86* | |
| Motion-confounds | *F(4, 92): 52986.3* | *0.75* | | | *< 0.001* | *1.00* | | *F(4, 92): 18.0* | | | *Sing.* | | *< 0.001* | *0.44* | | | | *F(4, 92): 6.8* | *Sing.* | | *< 0.001* | | *0.23* | |

*
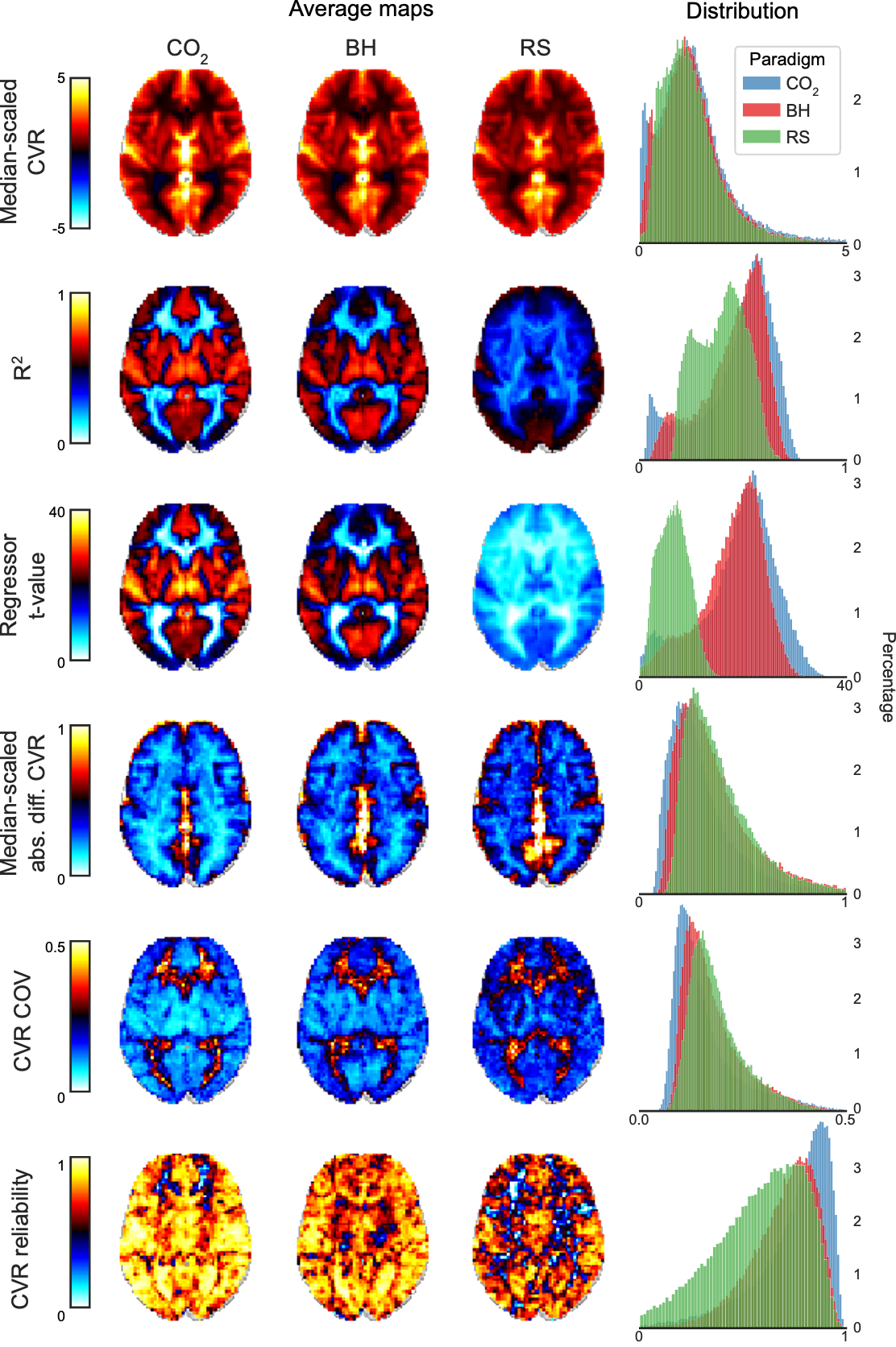
*

***Figure S9.*** *Average CVR and quality metric maps from using the best sets of design parameters (linear detrend order, low-pass cut-off frequency, confound-regressor correlation threshold) in terms test-retest repeatability (see Figure 5, main text). The CVR maps and CVR absolute difference maps have been scaled by the median CVR values across voxels, subjects and runs to compute comparable maps. Only voxels with a valid mask in at least 50% times across runs and subjects are included. Also shown in the distribution of voxel-wise values for each map.*

# Regressor versus CVR Map Repeatability


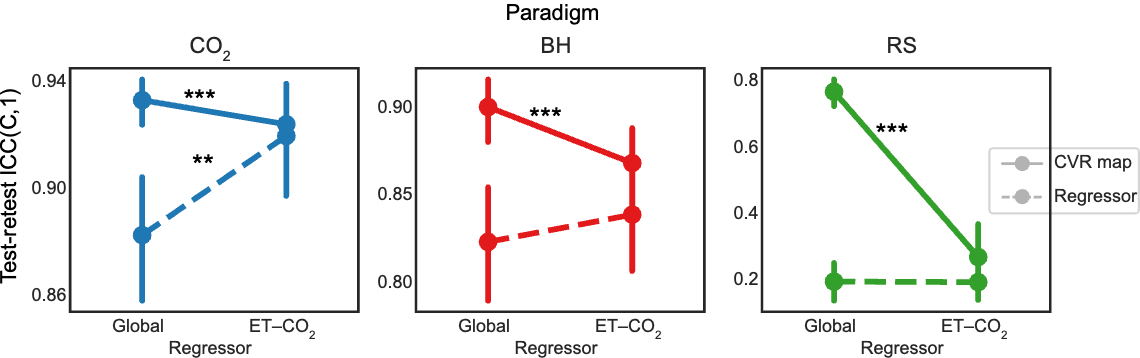


***Figure S10.*** *Effect of regressor choice (global signal or ET-CO_2_) on repeatability. Comparing test–retest ICC(C,1) for the regressor (dashed lines) and resulting CVR maps (solid lines) across the CO₂, BH, and RS paradigms.*

***Table S9.*** *Results from regressor repeatability analysis, using t-test and Shapiro-Wilk test for normality. Shown are the mean and standard deviation of the difference in repeatability of the regressor (ET-CO_2_ – global signal) and the resulting CVR maps, after Fisher’s z-transform, along with Shapiro-Wilk p-value (value larger than 0.05 signifies normality assumptions not broken), the t-statistic and degrees of freedom, corresponding p-value and effect size (Cohen’s d).*

| Paradigm | Repeatability | Mean difference | STD difference | Shapiro-Wilk p-value | t(df) | p-value | Cohen’s d |
| --- | --- | --- | --- | --- | --- | --- | --- |
| CO_2_ | *Regressor* | 0.25 | 0.34 | 0.43 | t(23) = 3.6 | 0.0016 | 0.73 |
|  | *CVR map* | -0.052 | 0.066 | 0.66 | t(23) = -3.8 | < 0.001 | -0.79 |
| BH | *Regressor* | 0.07 | 0.32 | 0.053 | t(23) = 1.05 | 0.30 | 0.21 |
|  | *CVR map* | -0.15 | 0.15 | 0.41 | t(23) = -4.9 | < 0.001 | -1.0 |
| RS | *Regressor* | -0.002 | 0.20 | 0.17 | t(23) = -0.059 | 0.95 | -0.012 |
|  | *CVR map* | -0.76 | 0.32 | 0.81 | t(23) = -11 | < 0.001 | -2.3 |

***Table S10.*** *Difference in average whole-brain CVR between runs (retest – test), using t-test and Shapiro-Wilk test for normality. Shown are the mean and standard deviation of the difference for the different paradigms and regressors, along with Shapiro-Wilk p-value (value larger than 0.05 signifies normality assumptions not broken), the t-statistic and degrees of freedom, corresponding p-value and effect size (Cohen’s d).*

| Paradigm | Regressor | Mean difference | STD difference | Shapiro-Wilk p-value | t(df) | p-value | Cohen’s d |
| --- | --- | --- | --- | --- | --- | --- | --- |
| CO_2_ | *ET-CO_2_* | -0.0077 | 0.017 | 0.95 | t(23) = -2.2 | 0.036 | -0.46 |
|  | *Global BOLD* | -0.0026 | 0.012 | 0.69 | t(23) = -1.1 | 0.28 | -0.22 |
| BH | *ET-CO_2_* | -0.0020 | 0.040 | 0.012 | t(23) = -0.24 | 0.81 | -0.05 |
|  | *Global BOLD* | 0.0037 | 0.022 | 0.39 | t(23) = 0.80 | 0.43 | 0.16 |
| RS | *ET-CO_2_* | -0.015 | 0.071 | 0.61 | t(23) = -1.1 | 0.30 | -0.22 |
|  | *Global BOLD* | 0.0070 | 0.029 | 0.0049 | t(23) = 1.2 | 0.24 | 0.25 |

In the two cases in Table S10 where the Shapiro–Wilk test indicated non-normality, Q–Q plots were inspected and showed no substantial deviations from normality. Therefore, no additional data transformations were deemed necessary.

# Median Filtering of Delay Maps

Applying a median filter (kernel size 3x3x3) to delay values prior to computing CVR values largely removed anti-correlated voxels (Figure S10). Here the median filter was applied to all voxels, a more sophisticated method could use information about the signal-regressor fit, as well as the autocorrelation structure of the regressor, to decide which voxels need filtering, such an approach is implemented in the advance package RapidTide (5).


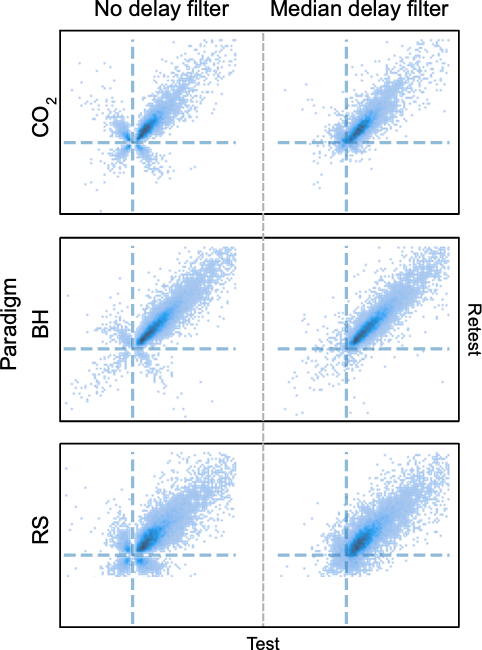


***Figure S10.*** *Effect of median filtering delay values prior to CVR estimation for a single subject (same as shown in the left column of Figure 6, main text). The left column displays voxel-wise test-retest CVR values when no median filtering is applied to the regressor delay values. The right column shows results after applying a 3×3×3 median filter, which reduces the occurrence of anti-correlated voxels. Delay bounds for alignment were set to: CO₂ = unbounded, BH = ±15 s, RS = ±8 s.*

# References

1. Cohen AD, Jagra AS, Visser NJ, Yang B, Fernandez B, Banerjee S, et al. Improving the Breath-Holding CVR Measurement Using the Multiband Multi-Echo EPI Sequence. Front Physiol. 2021 Feb 26;12:619714.

2. Cohen AD, Wang Y. Improving the Assessment of Breath-Holding Induced Cerebral Vascular Reactivity Using a Multiband Multi-echo ASL/BOLD Sequence. Sci Rep. 2019 Dec;9(1):5079.

3. Liu P, Hu B, Kartchner L, Joshi P, Xu C, Jiang D. Dependence of resting-state-based cerebrovascular reactivity (CVR) mapping on spatial resolution. Front Neuroimaging. 2023 June 26;2:1205459.

4. Ravi H, Thomas BP, Peng SL, Liu H, Lu H. On the optimization of imaging protocol for the mapping of cerebrovascular reactivity. J Magn Reson Imaging. 2016;43(3):661–8.

5. Frederick B deB, Salo T, Daniel M. Drucker PhD, Halchenko Y, Markiewicz C, Monroe D, et al. bbfrederick/rapidtide: Version 3.1.1 - 11/17/25 CVR output bug fix [Internet]. Zenodo; 2025 [cited 2025 Nov 18]. Available from: https://zenodo.org/records/17633117
